# *In silico* analysis for the development of multi-epitope vaccines against *Mycobacterium tuberculosis*

**DOI:** 10.3389/fimmu.2024.1474346

**Published:** 2024-11-18

**Authors:** Jin-Seung Yun, A Reum Kim, Soo Min Kim, Eunkyung Shin, Sang-Jun Ha, Dokeun Kim, Hye-Sook Jeong

**Affiliations:** ^1^ Korea National Institute of Health, Korea Disease Control and Prevention Agency, CheongJu, Republic of Korea; ^2^ Department of Biochemistry, College of Life Science and Biotechnology, Yonsei University, Seoul, Republic of Korea; ^3^ Chemicals Research Division, National Institute of Environmental Research, Incheon, Republic of Korea

**Keywords:** tuberculosis (TB), peptide-based vaccine, immunoinformatics analysis, multi-epitope, adjuvanted vaccine

## Abstract

As Bacille Calmette-Guérin (BCG) vaccine’s effectiveness is limited to only children, the development of new tuberculosis (TB) vaccines is being studied using several platforms, and a novel TB vaccine that overcomes this limitation is required. In this study, we designed an effective multi-epitope vaccine against *Mycobacterium tuberculosis* using immunoinformatic analysis. First, we selected 11 highly antigenic proteins based on previous research: Ag85A, Ag85B, Ag85C, ESAT-6, MPT64, Rv2660c, TB10.4, HspX, GlfT2, Fas, and IniB. Among these antigens, 10 linear B-cell epitopes, 9 helper T-cell epitopes, and 16 cytotoxic T-cell epitopes were predicted to design the multi-epitope vaccine. To improve the immunogenicity of the candidate vaccine, three different adjuvants, griselimycin, human beta-defensin 3 (HBD3), and 50s ribosomal protein (50sRP), were attached with linker sequences to the vaccine model. The immunogenic, antigenic, allergenic, and physicochemical properties of the resulting designed multi-epitope vaccines were predicted *in silico*. Moreover, 3D structural modeling, refinement, and validation were used to select a model for further evaluation. Molecular docking analysis revealed a consistent and significant binding affinity of the candidate vaccine for toll-like receptors (TLRs), TLR-2, -3, and -4. Immune simulation performed using C-ImmSim demonstrated that three rounds of immunization with multi-epitope vaccines induced a high production of cytokines and immunoglobulins related with both cellular and humoral immune response. Moreover, we constructed vaccine candidate composed of 50sRP and evaluated its immunogenicity in a mouse model. Consequently, this *in silico*-engineered multi-epitope structure can elicit adaptive immune responses and represents a promising novel candidate for TB vaccine development.

## 1 Introduction

Tuberculosis (TB), a highly contagious disease, is one of the most prominent causes of death worldwide and was the leading cause of death from a single infectious agent before the coronavirus disease (COVID-19) pandemic. According to the World Health Organization 2023 report, TB caused by *Mycobacterium tuberculosis* was projected to infect approximately one-quarter of the global population and kill approximately 1.5 million individuals in 2022 (1).

Once *M. tuberculosis* bacteria are inhaled, bacilli are primarily encountered in alveolar macrophages (AMs) located in the airway and migrate to the lung interstitium through host IL-1β signaling and the *M. tuberculosis* type VII secretion system, ESX-I (2). After entering the lung interstitium, the bacilli infect additional macrophages including monocyte-derived- or lung resident cells. Circulating dendritic cells (DCs) migrate to the draining lymph nodes, where they present antigen peptides bound to major histocompatibility complex (MHC) molecules on their surface to prime antigen-specific T cells. The MHC-antigen peptide complex interacts with the T cell receptor (TCR) on the surface of T cells (3). Upon binding of the TCR to the MHC-antigen peptide complex, antigen recognition signals are transmitted into the T cell, initiating its activation. Once activated, T cells differentiate into effector cells that secrete a variety of cytokines. In the case of *M. tuberculosis*, Th1 cells, which differentiated from CD4^+^ T cells, are the primary immune responders. They secrete cytokines such as IFN-γ, TNF-α, and IL-2, which recruit monocytes and neutrophils, enhance macrophage cytotoxicity, and induce the production of inflammatory mediators and reactive oxygen and nitrogen species, leading to the elimination of *M. tuberculosis*.

An effective TB vaccine relies on the generation of long-lived memory T cells, necessitating the activation of B- and T-cells. A multi-epitope vaccine, binding to MHCs, stimulates CTLs and HTLs by engaging TLRs to activate key immune components (4–6). B-cell epitopes are also vital in triggering memory immune responses through antibody production. Identifying specific epitope regions is crucial for predicting immune responses (7). Compared to a single subunit vaccine, the multi-epitope vaccine has an advantage in inducing immune response, especially considering the limited efficacy of single-subunit TB vaccines in humans due to the disease’s complex progression (6).

Vaccinating individuals against TB faces ongoing challenges because Bacillus Calmette-Guérin (BCG), the only approved TB vaccine, has limited efficacy in adults and adolescents (8, 9). To overcome the limitations of BCG, novel TB vaccines have been developed either as a booster to the current BCG vaccine or as prime vaccines to replace it. The protection provided by BCG is lacking in adults, which may be the result of waning immunity during childhood, leading to a deficiency in immunological memory (10). According to the report released by the WHO, 17 TB vaccine candidates are in the clinical trial stage (1). The most promising vaccine, M72/AS01_E_ which is comprised of Mtb32A and Mtb39A combined with the AS01_E_ adjuvant, showed good protection in healthy adults (11, 12) and HIV-infected adults (13). A Phase II b trial of M72/AS01_E_ showed 54.0% protective efficacy without substantial safety concerns (14). Furthermore, its effectiveness was 49.7% after three years of follow-up in final analyses of the efficacy, safety, and immunogenicity (15). However, the findings need to be confirmed over a longer period and in a larger population with different age groups and ethnicities. Therefore, studies for the development of more effective TB vaccines are a continuous endeavor.

In the realm of development of a TB vaccine, subunit vaccines utilizing specific antigens, such as M72/AS01E, H56:IC31, and ID93 + GLA-SE, which are currently under investigation in various clinical trials, offer the advantage of targeted and efficient development. However, their potential protective range might be limited due to the specificity of the selected antigens. In contrast, the multi-epitope TB vaccine strategy, anchored on a diverse set of antigens, holds promise for enhanced protection and broader efficacy. Bacterial pan-genomics analysis is recently being utilized to identify core genomes and potential vaccine targets in the development of multi-epitope-based vaccine candidates (16, 17). A proteomic analysis approach was also attempted, which utilized 242 virulent factors, 18 membrane proteins, 10 repair proteins, and 8 secretory proteins as potential candidates (18). For a comprehensive understanding of the approach to select pivotal antigens for the TB vaccine, comparing this method with other research endeavors that have evaluated *M. tuberculosis* vaccine candidates using various techniques is expected to be beneficial.

Additionally, ongoing research is harnessing various bioinformatics strategies for the judicious selection of epitope sequences. Investigations have been conducted to select effective MHC epitope regions by analyzing alleles and haplotypes that cover more than 95% of the world population (19), predict epitope sites in the RD1 region to overcome the limitations of the BCG vaccine (20), and use structural vaccinology tools to identify epitope sites that exhibit stability and antigenic tendencies (21). Such diverse approaches have advanced the design of antigen and epitope sequences. However, *M. tuberculosis* possesses an extensive array of over 4,000 antigens, and there remain unexplored areas with regard to its mechanisms of immune evasion and infection within the host.

To overcome the limitations of the BCG vaccine, next-generation TB vaccine or BCG-booster vaccines have been studied and developed using various platforms. Among them, peptide-based vaccines have advantages compared with traditional subunit vaccines. Although subunit vaccines are poor inducers of T-cell responses, peptide-based vaccines induce a more robust immune response with dominant epitope regions and reduce side effects caused by eliminating unwanted material from full-length protein (4, 22). Generally, peptide-based vaccines are designed by identification with bioinformatic tools and validated by using ELISpot in healthy and donor PBMCs (23). However, in the case of TB, there are numerous unrevealed TB antigens and their functions are largely unknown. Consequently, we have selected the Ag85 complex, ESAT-6, MPT64, Rv2660c, TB10.4, and HspX, as antigens from among the virulence factors *of M. tuberculosis* and those acting as immunogens within the host, with their vaccine efficacy validated through *in vivo* studies using animal models or clinical trials (24–34). Also, we include the antigens GlfT2, Fas, and IniB, based on a previous antigen identification study (35). Bettencourt et al. identified and studied antigens presented by MHC class I and II molecules in infected macrophages (35). The vaccine candidates, which were constructed with adenoviral vectors, carried respective antigens and showed immunogenicity and protective effectiveness as a BCG-booster vaccine. Furthermore, we predicted and validated their possibilities of use with its adjuvants, griselimycin, HBD3, and 50sRP, by using various bioinformatic tools. We selected the most soluble and the lowest Gibbs free energy model, which was best suited for TLR4 binding, and confirmed its immunogenicity in BCG-primed mice through *in vitro* expression and *in vivo* analysis.

## 2 Materials and methods

### 2.1 Antigen selection for vaccine preparation

Antigens were selected based on those included in the ongoing clinical trials and the results of another previous study (**Table 1**) (24, 25, 35–40). Ag85A, Ag85B, ESAT-6, Rv2660c, TB10.4, and HspX were constructed as subunit or virus vector vaccines and their effectiveness has been verified in animal models. Some antigens have progressed to the clinical trial phase in healthy adults and patients with TB (24, 36–39). Ag85C and MPT64 were reported as immunodominant antigens and are being investigated as vaccine candidates in several studies (25, 40). Additionally, the GlfT2, Fas, and IniB antigens were selected based on the results of another previous study (35). These antigens were immunoprecipitated with the MHC-I and MHC-II from human macrophage cells and their effectiveness has been evaluated in an animal model. Amino acid sequences of all antigens are acquired from Mycobrowser (https://mycobrowser.epfl.ch).

**Table 1:** Information on the selected antigens for multi-epitope vaccine construction.

Protein or Gene	Accession No.	Function or characteristic	Antigenicity	Allergenicity	Clinical stages (Candidate)	References
Ag85A	P9WQP2	Major immunodominant antigen	Antigen	Non-allergen	Phase 1 and 2a (AdHu5Ag85A, ChAdOx1.85A+MVA85A)	(26–28)
Ag85B	P9WQP1	Mycolyl transferase enzyme, Adhesive to macrophage	Antigen	Non-allergen	Phase 2b (H56:IC31)	(24, 36, 37)
Ag85C	P9WQN9	Cell envelope biogenesis	Antigen	Non-allergen	–	(25)
ESAT-6	P9WNK7	Secretory antigen, Virulence factor	Antigen	Non-allergen	Phase 2b (H56:IC31)	(24, 29, 36, 37)
MPT64	P9WIN9	Inhibition of apoptosis	Antigen	Allergen	–	(30, 31, 40)
Rv2660c	I6Y1F5	Latency-associated antigen	Antigen	Allergen	Phase 2b (H56:IC31)	(24, 32, 36, 37)
TB10.4	P9WNK3	Function unknown. May be involved in virulence	Antigen	Non-allergen	Phase 1 (TB/FLU-05E)	(33, 38, 39)
HspX	P9WMK1	Regulation of *M. tuberculosis in vivo* growth	Antigen	Allergen	Phase 1 (TB/FLU-05E)	(34, 38, 39)
GlfT2	O53585	Formation of mycobacterial cell wall, Biosynthesis of galactan chain	Antigen	Non-allergen	–	(35, 125)
Fas	P95029	Fatty acid synthase	Antigen	Non-allergen	–	(35)
IniB	P9WJ97	Unknown	Antigen	Non-allergen	–	(35)

### 2.2 Predicting linear B-cell epitopes

Among the various tools for B-cell epitope prediction, including BcePred (41) and BepiPred (42), ABCPred distinguishes itself by employing Artificial Neural Networks (ANN) (43). While BcePred relies on physicochemical properties and BepiPred utilizes a combination of the hidden Markov model and propensity scale method (41, 42), ABCPred harnesses the capabilities of ANN to detect patterns without predefined rules and this approach allows ABCPred to learn more complex interactions from the data achieving an accuracy of 65.93% (43). The ABCpred server (http://www.imtech.res.in/raghava/abcpred) was used to identify linear B-cell epitopes. The prediction process involved using the default threshold of 0.51, and a score > 0.87 was required for the 16-mer epitopes. Among the 11 antigens, Ag85A was removed from the analysis since Ag85A and Ag85B had identical amino acid sequences. A higher score indicates a probability of being a B-cell epitope. Therefore, we selected the final epitope sequence based on the highest score.

### 2.3 Predicting helper T lymphocyte epitopes

HTL epitopes serve play a pivotal role in coordinating the immune defense against intracellular pathogens such as M. tuberculosis. They act as a crucial link between innate immune recognition and the activation of cytotoxic responses, ensuring a targeted and effective immune reaction. To predict HTL epitope sequence, the Immune Epitope Database (IEDB) MHC-II server was utilized to identify human leukocyte antigen (HLA) class II epitopes, also known as HTL epitopes (44, 45). The species/locus was chosen as Human/HLA-DR, and a 7-allele HLA reference set (HLA-DRB1*03:01, HLA-DRB1*07:01, HLA-DRB1*15:01, HLA-DRB3*01:01, HLA-DRB3*02:02, HLA-DRB4*01:01, HLA-DRB5*01:01) was selected for the HTL epitope prediction. The server predicted the binding affinity of 15-mer peptides and generated percentile scores for each peptide based on its predicted binding affinity relative to a large set of random peptides. The peptides were then categorized based on their percentile scores, with those having higher scores considered to have stronger predicted binding affinity to the HLA class II allele (HLA-DR). Finally, the HTL epitopes were selected based on both their percentile scores and predicted ability to induce the production of IFN-γ, a cytokine involved in the immune response.

### 2.4 Predicting IFN-γ immune response-inducing epitopes

To determine the capacity of the predicted HTL epitopes to induce an IFN-γ immune response, they were analyzed using the IFNepitope server (http://crdd.osdd.net/raghava/ifnepitope/) (46). The server calculates an IFN-γ score for each peptide based on a hybrid approach that combines a motif approach and support vector machine (SVM) approach (46, 47). Through this hybrid approach, positive-scoring epitopes were selected as IFN-γ-inducible epitopes based on their IFN-γ scores. We selected the epitope sequence with the highest IFN-γ score to maximize its potential for inducing IFN-γ production, thereby anticipating a stronger immune response.

### 2.5 Predicting cytotoxic T lymphocyte epitopes

In immune response against *M. tuberculosis* infection, cytotoxic T lymphocyte attributes cytolytic action against pathogen by means of cell-to-cell contact determines the apoptosis of the pathogen infected macrophages (48, 49). Predictions of the 9-mer CTL epitopes were conducted using the NetCTL 1.2 server (http://www.cbs.dtu.dk/services/NetCTL) and based on A1 supertypes, which are commonly found in humans (50). The analysis included MHC-I binding affinity, C-terminal cleavage affinity, and efficiency of antigen processing transport (51) and each parameter was set 0.75, 0.15, and 0.05, respectively.

### 2.6 Designing multi-epitope vaccine sequence candidates

Based on the analyses described, high-scoring B-cell, CTL, and HTL epitopes were chosen to design a vaccine candidate with strong antigenicity and immunogenicity, and low toxicity. The linear B-cell and HTL epitopes were attached with GPGPG sequences, while the CTL epitopes were attached with AAY sequences. Griselimycin (52, 53), human β-defensin 3 (HBD3) (54, 55), and 50s ribosomal protein (50sRP) (56) were selected as adjuvants to enhance vaccine immunogenicity and were added to both the N- and C-terminals via EAAAK sequences (7, 52). This adjuvant sequence was sourced from the UniProt database (http://www.uniprot.org/).

### 2.7 Predicting multi-epitope vaccine sequence as inducers of pro- or anti-inflammatory cytokines

The potential immunogenicity of the final sequence for the induction of cytokines, IL-4, IL-6, and IL-10, was systematically assessed. This assessment employed specialized web servers, including IL4Preb, IL6Preb, and IL10Preb (57–60). For IL-4 induction, a hybrid analytical approach integrating SVM with Motif was adopted. Peptides were designated as IL-4 inducers based on a predetermined threshold exceeding 0.2. For predictions for IL-6 and IL-10 secretion, an array of machine-learning methodologies was explored. Notably, the Random Forest-based model showed superior predictive accuracy for IL-6 secretion. Peptides were discerned as inducers for IL-6 and IL-10 based on threshold values exceeding 0.11 and -0.03, respectively (61, 62).

### 2.8 Predicting antigenicity and allergenicity

The antigenicity of each candidate multi-epitope vaccine attached to its respective adjuvant was predicted using two online tools: VaxiJen 2.0 (http://www.ddg-pharmfac.net/vaxijen/VaxiJen/VaxiJen.html) (63) and the ANTIGENpro server (http://scratch.proteomics.ics.uci.edu) (64). VaxiJen 2.0 uses autocross covariance (ACC) transformation of protein sequences to determine antigenicity (63), while ANTIGENpro is a sequence-based, pathogen-independent tool for predicting antigenicity (64). ToxinPred server (http://crdd.osdd.net/raghava/toxinpred/) were employed to predict toxicity of multi-epitope sequence. This server facilitates the design of toxic peptides and the identification of toxin regions within proteins (65). AllerTOP v2.0 (http://www.pharmfac.net/allertop) and AllergenFP (http://ddg-pharmfac.net/AllergenFP/) were employed to predict the allergenicity of each of the vaccine candidates. AllerTOP v2.0 is a server that uses the physicochemical properties of proteins to predict allergens and is the first alignment-free tool of its kind. AllergenFP is an alignment-free method that predicts allergenicity based on properties such as hydrophobicity, size, relative abundance, helix formation, and β-strand forming tendencies of the amino acids (66).

### 2.9 Predicting physicochemical properties and solubility

The Expasy ProtParam server (https://web.expasy.org/protparam) was used to predict various physicochemical properties of the final multi-epitope vaccines. These properties include the grand average of hydropathicity (GRAVY), aliphatic index, *in vitro* and *in vivo* half-lives, instability index, theoretical isoelectric point (pI), and molecular weight. In addition, the Protein-Sol server (http://protein-sol.manchester.ac.uk) was utilized to predict the solubilities of the multi-epitope vaccine candidates (67). The predicted solubility (QuerySol) was represented as a scaled solubility value, where any value above 0.45 was considered to have higher solubility than the average value (PopAvrSol) from the experimental dataset. Conversely, proteins with lesser-scaled solubility values were considered to have lower solubility (67).

### 2.10 Predicting secondary and tertiary structure

To predict the secondary structures of the multi-epitope vaccines designed with their respective genetic adjuvants, we used the PSIPRED (Position-Specific Iterated PREDiction) protein structure prediction server (http://bioinf.cs.ucl.ac.uk/psipred). The PSIPRED is a highly accurate method for predicting secondary structure, with an average Q3 score of 76.5%, suggesting that it is a reliable tool for determining the secondary structures of the multi-epitope vaccine candidates (68). Furthermore, the RaptorX Property web server (http://raptorx.uchicago.edu/StructurePropertyPred/predict/) was used to predict the secondary structure properties of the multi-epitope vaccine in the absence of a template. This server is designed to predict various structural properties of a protein using a deep-learning model. Hence, it is a useful tool for predicting secondary structure properties.

Then, the I-TASSER (Iterative Threading ASSEmbly Refinement) server (https://zhanglab.ccmb.med.Umich.Edu/I-TASSER/) was utilized to construct the tertiary models of the multi-epitope vaccines. This server follows a sequence-to-structure-to-function approach, which involves predicting and identifying similar structural patterns from the Protein Data Bank (PDB) to create a 3D model. To assess the accuracy of the generated models, the server provides a template modeling (TM) score. A TM score greater than 0.5 indicates a reliable topological model, whereas a TM score less than 0.17 suggests a random similarity. These TM score cut-off values are not dependent on the length of the protein being modeled (69). Therefore, these TM score cut-offs could be used to evaluate the accuracy of the tertiary models of the multi-epitope vaccines generated by the I-TASSER server. The confidence scores (C-scores) calculated by I-TASSER typically range from -5 to 2, where a higher C-score indicates higher accuracy (69).

### 2.11 Refinement of the tertiary structure

To further improve the accuracy of the generated tertiary model of the multi-epitope vaccines, the GalaxyRefine server (http://galaxy.seoklab.org/cgi-bin/submit.cgi?type=REFINE) was used to refine the model. The GalaxyRefine method combines side-chain repacking followed by molecular dynamics simulations to relax the overall structure (70). GalaxyRefine is effective in improving the local structure quality, as demonstrated by the results of the CASP10 assessment. The quality of the refined model was evaluated based on several metrics, including GDT-HA, root-mean-square deviation (RMSD), MolProbity, clash, and Ramachandran plot scores. These metrics are commonly used to assess the accuracy and quality of protein models and provide a comprehensive evaluation of the refined model’s structural features.

### 2.12 Validation of the tertiary structure

Several web servers were used to assess and validate the reliability of the refined tertiary structures. First, the ProSA web server (https://prosa.services.came.sbg.ac.at/prosa.php) was used to determine the overall quality score for the input structure. A calculated score outside the range characteristic of native proteins suggests the presence of errors in the structure. Next, the ERRAT server (http://services.mbi.ucla.edu/ERRAT/) was utilized to compare the non-bonded atom-atom interactions with those of reliable high-resolution crystallographic structures, helping identify any potential errors or inaccuracies in the model. Finally, the RAMPAGE web server (http://mordred.bioc.cam.ac.uk/~rapper/rampage.php) was used to generate a Ramachandran plot (71). The RAMPAGE output includes the percentages of residues in the allowed and disallowed regions, providing information about the overall quality of the modeled structure. These validation steps provide confidence in the accuracy and reliability of the final refined tertiary model of the multi-epitope vaccine (72).

### 2.13 Predicting discontinuous B-cell epitopes

To identify the discontinuous B-cell epitopes in the refined final 3-D structure models, we used the ElliPro server (http://tools.iedb.org/ellipro). ElliPro employs a geometry-based approach to analyze protein structure and predict B-cell epitopes based on their spatial arrangement. The server provides an AUC (Area Under the Curve) value of 0.732, which is considered the best calculation out of all proteins (73). By using ElliPro, we were able to predict the locations of the discontinuous B-cell epitopes in the refined final model of the multi-epitope vaccine.

### 2.14 Molecular docking analysis of the final vaccine with toll-like receptors

The designed multi-epitope vaccines were subjected to molecular docking analysis with TLR2, TLR3, and TLR4 (PDB ID:1ZIW, 3A7B, 4G8A) using the HADDOCK 2.4 web server (http://bianca.science.uu.nl/haddock2.4), predicting the appropriate immune response and interactions between the final vaccine structures and these specific immune receptors (74). The HADDOCK 2.4 web server employs an integrative approach that combines biochemical and biophysical information with computational modeling to predict protein-protein interactions. The server calculates the lowest energy score for the final docking complex, which is indicative of the most stable complex (75). By using HADDOCK 2.4, the binding affinity of the multi-epitope vaccine to TLR2, TLR3, and TLR4 was predicted, a process that can help in designing effective vaccines.

### 2.15 Normal mode analysis

To better understand the stability and dynamicity of the interactions of the final vaccine structures with the TLR4 receptors, a normal mode analysis was performed. The iMODS web server (http://imods.Chanconlab.org/) was used for this purpose, as it allows for the analysis of protein flexibility and conformational changes over time (76). The simulation results were analyzed in terms of deformability, eigenvalue, b-factor, variance, correlation matrix, and the elastic network model.

### 2.16 Codon optimization and *in silico* cloning

The Java Codon Adaptation Tool (JCat) server (http://www.prodoric.de/JCat) was employed to improve protein expression efficiency via codon optimization (77). JCat is an adjustable tool that tailors the codon usage of an input sequence to better suit specific organisms, enhancing expression levels; in this study, it was used to adjust codon usage for *E. coli*. Following optimization, the codon-optimized sequence was cloned into the *E. Coli* pET30a (+) vector using the Snapgene 7.0.2 tool (https://snapgen.com/).

### 2.17 Immune simulation

Finally, the C-ImmSim server (http://150.146.2.1/C-IMMSIM/index.php) was used for *in silico* immune simulations to assess the immunogenicity and immune response generated by the multi-epitope vaccines. C-ImmSim is an agent-based model that utilizes position-specific scoring matrices for peptide prediction derived from machine-learning techniques (78). The default simulation parameters were used, with time steps set at 1, 84, and 168. Each time step represents 8 h, with the first injection given at time = 0, followed by two more injections at twelve-week intervals (79). The simulations aimed to characterize the immune interactions and evaluate the efficacy of the multi-epitope vaccines.

### 2.18 Cloning the designed gene and expression and purification of the recombinant protein

To construct the *in silico* designed recombinant protein, the nucleotide sequence of 50sRP, verified by *in silico* cloning, along with the addition of a poly histidine-tag (6x his tag) at the C-terminus was synthesized and cloned into a commercial pET-30a (+) expression vector (80). The recombinant expression vector was transformed into *E. Coli* BL21 (ED3) cells. A single colony was inoculated into the LB medium containing kanamycin. Expression of the recombinant protein was induced by adding isopropyl-β-thiogalactopyranoside (IPTG; 0.5mM) and assayed by using SDS-PAGE and western blotting using an anti-His antibody (Genscript, Piscataway, NJ, USA) at dilution of 1:1000. The expressed recombinant protein was purified with an Ni-NTA column under denaturing conditions following the manufacturer’s instructions (81). The purified protein was dialyzed using a urea solution and phosphate-buffered saline (pH 7.2) overnight at 4°C.

### 2.19 Animals, ethics statement and immunization

Four to five-week-old female C57BL/6 mice were purchased from Samtako (Seoul, Korea). Mice were kept under standard environmental conditions with commercial food and tap water (both ad libitum). Mice were acclimated at facility during 7 days. After 1 week, five to six-week-old mice (n = 5) were vaccinated with PBS or BCG Pasteur 1173P2 (2 × 10^5^ CFUs/mouse) subcutaneously (week 0). After 6 weeks, BCG-primed mice were boosted with vaccine candidate; 50sRP-TB (5 μg/mouse) twice at an interval of 3 weeks (1^st^ boost: 6 weeks, 2^nd^ boost: 9 weeks). One week after the final immunization (at 10 weeks), the mice (n = 5) were euthanized via CO_2_ inhalation to analyze immunogenicity.

### 2.20 Preparation of *M. bovis* BCG

*Mycobacterium bovis (M. bovis)* BCG Pasteur 1173P2 were provided by the Korea Disease Control and Prevention Agency (KDCA) and cultured in Middlebrook’s 7H9 broth (Difco Laboratories, Detroit, MI) supplemented with 10% Oleic Albumin Dextrose Catalase (OADC) enrichment (Becton Dickinson, Sparks, MD) and 0.2% glycerol at 37°C on a shaker at 200 rpm under aerobic conditions for 14–20 d (82). To obtain single-cell suspensions, mycobacterial cell culture media were centrifugated at 10,000 × *g* for 20 min and washed thrice with PBS. The pellet was resuspended in PBS supplemented with 0.05% tyloxapol (Sigma Aldrich, Saint Louse, MO) to prevent clumping and passed through 40-, 20-, 10-, and finally, 8-μm filters (Millipore Corp., Burlington, MA, USA). The final stock was stored in small aliquots at −80°C until further use. Colony-forming units (CFUs) per milliliter of stock were measured using a counting assay on 7H10 agar plates (Difco Laboratories).

### 2.21 Preparation of single lymphocyte cells

Five mice from each group were sacrificed 1 week after the last immunization. Lung and spleen were harvested aseptically from euthanized mice and single cells extracted. To isolate lung lymphocyte, tissue was incubated with 40U/ml DNase I (Roche, Swiss) and 2mg/ml collagenase D (Roche) in a plain RPMI-1640 medium (GenDEPOT, TX, USA) at 37°C for 1 h. Enzyme-treated lung tissue and spleen were homogenized using a gentleMACS μTissue Dissociator (Miltenyl Biotec, Germany), washed in RPMI-1640 medium (GenDEPOT, TX, USA), and supplemented with 10% FBS. Spleen was rinsed in ammonium-chloride-potassium buffer to remove erythrocytes. Lung lymphocytes were separated using Lymphoprep (STEMCELL Technologies, Canada) with density centrifugation and resuspended in RPMI containing 10% fetal bovine serum (FBS, Gibco, MA, USA) and 1% penicillin/streptavidin (P/S, Gibco).

### 2.22 Antigen specific enzyme-linked immune spot

The ELISpot assay was performed using an IFN-γ secretion ELISpot kit. Briefly, a single-cell suspension (5 × 10^5^ cells) was stimulated with pooled multi-epitope peptides (100ng/mL each) for 36 h at 37°C in anti-IFN-γ antibody-coated filter plates. Afterward, biotinylated anti-IFN-γ antibody, streptavidin-horseradish peroxidase (HRP) conjugate, and 3-amino-9-ethylcarbazole were added as substrates to develop secreted cell spots, which were quantified using an Immunospot S6 analyzer (Cellular Immunospot Limited). The results are presented as mean values of triplicate wells for each group. All substrates and the ELISpot kits were purchased from Becton Dickinson.

### 2.23 Enzyme-linked immune sorbent assay

For the measurement of immunoglobulin titers, 96-well flat bottom Immuno Plates (Thermo Fisher Scientific, MA, USA) were coated with pooled epitope peptides (100ng/mL each) diluted with ELISA coating buffer (BioLegned, CA, USA) for 18 h at 4°C. The plates were washed thrice with PBS containing 0.5% Tween 20 and blocked with 5% skimmed milk (Difco Laboratories). After washing, the mouse serum samples were diluted at 1:200 with PBS containing 3% BSA and incubated for 2 h at 37°C. Subsequently, a 1:2,000 dilution of goat anti-mouse IgG-HRP (Thermo Fisher Scientific) was added and incubated for 1 h at 37°C. The substrate, tetramethylbenzidine (TMB; Thermo Fisher Scientific), was added to each well, and the plates were incubated at 37°C for 15–30 min. Thereafter, a stop solution for TMB was added, and the plates were read using a spectrophotometer (Spectramax i3x, Molecular Devices, CA, USA) at 450 nm.

### 2.24 Statistical analyses

To determine statistical significance, one-way Analysis of Variance (ANOVA) using Dunnett’s multiple comparison test (comparing to PBS immunized mice only) was used for evaluation of significant differences between more than two vaccine groups. The differences with a *p*-value <0.05 were considered significant. Data expressed in graphs are presented as mean ± standard deviation. All analyses were performed using GraphPad Prism v10 (GraphPad Software, CA, USA).

## 3 Results

### 3.1 Linear B-cell epitope prediction

The functional sequences of the eleven proteins were subjected to linear B-cell epitope prediction using the ABCpred server (**Table 2**). The 16 mer peptides of the Ag85B, Ag85C, ESAT-6, MPT64, Rv2660c, TB10.4, HspX, GlfT2, Fas, and IniB were selected for the final vaccine based on their binding scores (> 0.51) and highest predicted score (83).

**Table 2:** Predictions of linear B-cell epitopes, the highest predicted score epitope was selected for the final multi-epitope tuberculosis vaccine construct.

Serial no.	Gene	Protein	Peptide sequence	Start position	Predicted score (ABCpred)
1	Rv1886c	Ag85B(FbpB)	YSDWYSPACGKAGCQT	126	0.94
2	Rv0129c	Ag85C(FbpC)	NSMWGPSSDPAWKRND	230	0.9
3	Rv3875	ESAT-6(EsxA)	KWDATATELNNALQNL	77	0.89
4	Rv1980c	MPT64	AATSSTPREAPYELNI	96	0.92
5	Rv2660c	Uncharacterized	AAGASGGVTVGVGVGT	32	0.88
6	Rv0288	TB10.4	AWQGDTGITYQAWQAQ	62	0.93
7	Rv2031c	HspX	DEMKEGRYEVRAELPG	47	0.94
8	Rv3808c	GlfT2	QVHRIRKSYPDAVVLP	502	0.95
9	Rv2524c	Fas	TGLIRWEDDPQPGWYD	2495	0.97
10	Rv0341	IniB	TQPQHTPVEPPVHDKP	444	0.96

### 3.2 T-cell epitope prediction

Inducing CTL- and HTL-mediated immune responses is a critical step in vaccine design. Within eleven of the antigens, several epitope regions with high binding affinity for the human HLA-DR alleles (a 7-allele HLA reference set) were identified using the IEDB MHC-II server, allowing the selection of promising epitopes. As a result, nine peptide sequences distinguished by their robust IFN-γ induction potential and low percentile scores were incorporated into the final vaccine candidates to induce an HTL immune response (**Table 3**). Specifically, antigens Ag85C and MPT64 were excluded from the final antigen list because Ag85C had a similar amino acid sequence to that of Ag85B and MPT64 elicited an extremely low IFN-γ immune response (IFN-γ score: 0.2038). As lower percentile scores represent higher MHC-II binding affinity (84), we choose the final epitope region of each antigen showing the lowest percentile score. An SVM-based approach was used to examine the positive versus negative IFN-γ responses to identify epitope regions that induce an IFN-γ response.

**Table 3:** List of the helper T lymphocyte epitopes selected from the Immune Epitope Database (IEDB) MHC-Ⅱ server based on their high binding affinity for major histocompatibility complex (MHC)-Ⅱ human leukocyte antigen (HLA)-DR alleles.

Serial no.	Gene	Protein	Allele	Start	End	Peptide sequence	Percentile score	Method	Result	IFN-γ score
1	Rv3804c	Ag85A	HLA-DRB3*02:02	240	254	VGKLIANNTRVWVYC	0.83	SVM	Positive	0.70837188
2	Rv1886c	Ag85B	HLA-DRB3*02:02	239	253	KLVANNTRLWVYCGN	0.80	SVM	Positive	0.57095517
3	Rv3875	ESAT-6	HLA-DRB3*02:02	65	79	LNNALQNLARTISEA	8.50	SVM	Positive	0.81741208
4	Rv2660c	Uncharacterized	HLA-DRB1*03:01	28	42	VGVGVGTEQRNLSVV	17.00	SVM	Positive	0.69000995
5	Rv0288	TB10.4	HLA-DRB3*02:02	1	15	MSQIMYNYPAMLGHA	0.54	SVM	Positive	0.7187742
6	Rv2031c	HspX	HLA-DRB3*02:02	94	108	AYGSFVRTVSLPVGA	0.11	SVM	Positive	1.320439
7	Rv3808c	GlfT2	HLA-DRB1*15:01	3	17	ELAASLLSRVILPRP	8.20	SVM	Positive	0.54497781
8	Rv2524c	Fas	HLA-DRB5*01:01	358	372	IRGLGIGIVPAATRG	2.10	SVM	Positive	0.90807083
9	Rv0341	iniB	HLA-DRB3*02:02	257	271	NAVLASNASGQAGLI	2.20	SVM	Positive	0.4605373

The final 16 epitope regions of the 9 antigens were identified using the NetCTL 1.2 web server based on their MHC-I binding affinity, C-terminal cleavage affinity, and transport efficiency to select those that induce a CTL response. Notably, ESAT-6 and Ag85A were excluded from the final antigen list because ESAT-6 had an extremely low transport efficiency (-0.656) and Ag85A had a similar amino acid sequence to Ag85B. Therefore, we selected multiple sequences of Ag85B with higher affinity and efficiency scores instead (**Table 4**). The final epitope sequences were characterized based on the pro- or anti-inflammatory features. Using several immune-informative tools, IL-4, IL-6, and IL-10 inducibility of respective epitope sequence was predicted (**Table 5**). 6 B cell epitope, 5 HTL epitope, and 12 CTL epitope were predicted as IL-4 inducer, and 8 B cell epitope, 1 HTL epitope, 16 CTL epitope were predicted as IL-6 inducer. Taken together, a considerable proportion of the CTL epitope sequence was predicted as IL-4 and IL-6 inducers which were pro-inflammatory cytokines engaged in the regulation of *M. tuberculosis* (85–88). Only one, namely the HTL epitope of Rv3808c (GlfT2), was confirmed as an inducer of IL-10, known as the anti-inflammatory cytokine in the pathogenesis of Mycobacteria (**Table 5**).

**Table 4:** List of the cytotoxic T lymphocyte (CTL) epitopes selected from NetCTL 1.2 server based on their high binding affinity with MHC-Ⅰ A1-supertype alleles and antigenicity as well as low/no allergenicity and toxicity.

Serial no.	Gene	Protein	Peptide sequence	MHC binding affinity	Rescale binding affinity	C-terminal cleavage affinity	Transport efficiency	Prediction score
1	Rv1886c	Ag85B	QSSFYSDWY	0.6538	2.776	0.7522	2.944	3.036
2			NTPAFEWYY	0.6092	2.5865	0.9356	2.934	2.8735
3	Rv0129c	Ag85C	QSNGQNYTY	0.5528	2.347	0.9514	2.938	2.6366
4			GSALILAAY	0.3703	1.5722	0.9387	2.773	1.8516
5	Rv1980c	MPT64	DTDPLPVVF	0.2605	1.1059	0.9344	2.175	1.3548
6	Rv2660c	Uncharacterized	GTEQRNLSV	0.2676	1.1362	0.8093	0.017	1.2584
7	Rv0288	TB10.4	AMEDLVRAY	0.3203	1.3601	0.8827	2.997	1.6423
8			WQGDTGITY	0.2	0.849	0.9583	2.964	1.1409
9	Rv2031c	HspX	RSEFAYGSF	0.1537	0.6525	0.531	2.645	0.8644
10			DEDDIKATY	0.1204	0.5111	0.9728	2.451	0.7796
11	Rv3808c	GlfT2	WTAAPHAEY	0.6518	2.7675	0.9712	2.929	3.0596
12			NTDCQQILF	0.626	2.6579	0.6017	2.475	2.8719
13	Rv2524c	Fas	FSPAEVMRY	0.508	2.1568	0.8393	2.825	2.4239
14			LSGRWAQAY	0.4623	1.9629	0.8115	2.754	2.2223
15	Rv0341	IniB	TTDVGAGLA	0.3122	1.3256	0.1874	-0.745	1.3164
16			LIDYILSLF	0.2159	0.9167	0.692	2.545	1.1477

**Table 5:** *In-silico* characterization of multi-epitope sequence of TB vaccine model as pro- or anti-inflammatory.

	Gene	Sequence	Hybrid (SVM + Motif) method-based score of peptide being IL-4 inducer^a^ (Threshold 0.2)		Random forest method-based score of peptide being IL-6 inducer^b^ (Threshold 0.11)		SVM method-based score of peptide being IL-10 inducer^c^ (Threshold -0.3)	
B cell epitope	Rv1886c	YSDWYSPACGKAGCQT	IL-4 inducer	0.26	IL-6 inducer	0.16	IL-10 non-inducer	0.26860554
	Rv0129c	NSMWGPSSDPAWKRND	IL-4 inducer	0.30	IL-6 inducer	0.18	IL-10 non-inducer	-0.4494465
	Rv3875	KWDATATELNNALQNL	IL-4 inducer	0.74	IL-6 inducer	0.26	IL-10 non-inducer	-0.22631866
	Rv1980c	AATSSTPREAPYELNI	IL-4 non-inducer	-0.12	IL-6 inducer	0.12	IL-10 non-inducer	0.23738934
	Rv2660c	AAGASGGVTVGVGVGT	IL-4 inducer	0.22	IL-6 non-inducer	0.1	IL-10 non-inducer	-0.095451042
	Rv0288	AWQGDTGITYQAWQAQ	IL-4 inducer	0.28	IL-6 inducer	0.14	IL-10 non-inducer	-1.1234132
	Rv2031c	DEMKEGRYEVRAELPG	IL-4 non-inducer	-0.26	IL-6 inducer	0.2	IL-10 non-inducer	0.13524524
	Rv3808c	QVHRIRKSYPDAVVLP	IL-4 inducer	0.24	IL-6 inducer	0.24	IL-10 non-inducer	0.082145961
	Rv2524c	TGLIRWEDDPQPGWYD	IL-4 non-inducer	0.11	IL-6 non-inducer	0.05	IL-10 non-inducer	0.11818483
	Rv0341	TQPQHTPVEPPVHDKP	IL-4 non-inducer	0.20	IL-6 inducer	0.25	IL-10 non-inducer	-0.079992129
HTL epitope	Rv3804c	VGKLIANNTRVWVYC	IL-4 inducer	0.45	IL-6 non-inducer	0.06	IL-10 non-inducer	0.4094788
	Rv1886c	KLVANNTRLWVYCGN	IL-4 non-inducer	-0.02	IL-6 non-inducer	0.08	IL-10 non-inducer	0.14951792
	Rv3875	LNNALQNLARTISEA	IL-4 inducer	0.74	IL-6 non-inducer	0.03	IL-10 non-inducer	0.29902151
	Rv2660c	VGVGVGTEQRNLSVV	IL-4 inducer	0.52	IL-6 non-inducer	0.02	IL-10 non-inducer	0.13102067
	Rv0288	MSQIMYNYPAMLGHA	IL-4 inducer	0.21	IL-6 non-inducer	0.02	IL-10 non-inducer	-0.18040595
	Rv2031c	AYGSFVRTVSLPVGA	IL-4 inducer	0.51	IL-6 non-inducer	0.02	IL-10 non-inducer	-0.03682633
	Rv3808c	ELAASLLSRVILPRP	IL-4 non-inducer	0.14	IL-6 inducer	0.25	IL-10 inducer	1.0525806
	Rv2524c	IRGLGIGIVPAATRG	IL-4 non-inducer	0.13	IL-6 non-inducer	0.04	IL-10 non-inducer	-0.24677607
	Rv0341	NAVLASNASGQAGLI	IL-4 non-inducer	-0.19	IL-6 non-inducer	0.06	IL-10 non-inducer	-0.63953613
CTL epitope	Rv1886c (1)	QSSFYSDWY	IL-4 inducer	0.26	IL-6 inducer	0.76	IL-10 non-inducer	0.30883378
	Rv1886c (2)	NTPAFEWYY	IL-4 inducer	0.44	IL-6 inducer	0.81	IL-10 non-inducer	-0.35864931
	Rv0129c (1)	QSNGQNYTY	IL-4 non-inducer	0.10	IL-6 inducer	0.64	IL-10 non-inducer	-0.12903001
	Rv0129c (2)	GSALILAAY	IL-4 non-inducer	-0.10	IL-6 inducer	0.82	IL-10 non-inducer	0.006059691
	Rv1980c	DTDPLPVVF	IL-4 inducer	0.24	IL-6 inducer	0.76	IL-10 non-inducer	-0.15565183
	Rv2660c	GTEQRNLSV	IL-4 inducer	0.33	IL-6 inducer	0.83	IL-10 non-inducer	-0.55210576
	Rv0288 (1)	AMEDLVRAY	IL-4 inducer	0.14	IL-6 inducer	0.85	IL-10 non-inducer	0.008026008
	Rv0288 (2)	WQGDTGITY	IL-4 inducer	0.28	IL-6 inducer	0.8	IL-10 non-inducer	-1.1211936
	Rv2031c (1)	RSEFAYGSF	IL-4 inducer	0.41	IL-6 inducer	0.8	IL-10 non-inducer	-0.5152663
	Rv2031c (2)	DEDDIKATY	IL-4 inducer	0.28	IL-6 inducer	0.88	IL-10 non-inducer	-0.82231135
	Rv3808c (1)	WTAAPHAEY	IL-4 inducer	0.21	IL-6 inducer	0.6	IL-10 non-inducer	-0.13767462
	Rv3808c (2)	NTDCQQILF	IL-4 non-inducer	0.19	IL-6 inducer	0.85	IL-10 non-inducer	-0.33668965
	Rv2524c (1)	FSPAEVMRY	IL-4 non-inducer	0.11	IL-6 inducer	0.9	IL-10 non-inducer	-0.038967804
	Rv2524c (2)	LSGRWAQAY	IL-4 inducer	0.26	IL-6 inducer	0.83	IL-10 non-inducer	-0.65072028
	Rv0341 (1)	TTDVGAGLA	IL-4 inducer	0.24	IL-6 inducer	0.7	IL-10 non-inducer	-0.521025
	Rv0341 (2)	LIDYILSLF	IL-4 inducer	0.28	IL-6 inducer	0.88	IL-10 non-inducer	0.3457436

All the peptide sequences of 16 B cell epitope, 9 HTL epitope, and 10 CTL epitope were subjected to analysis by using three kinds of bio-informatics tools to characterize their pro- or anti-inflammatory nature.

a Prediction of peptide as an IL-4 inducer or non-inducer using IL-4pred.

b Prediction of peptide as an IL-6 inducer or non-inducer using IL-6pred.

c Prediction of peptide as an IL-10 inducer or non-inducer using IL-10pred. Among the sequences, 6 B cell, 5 HTL, and 12 CTL epitopes were predicted as IL-4 inducers. 8 B cell epitope, 1 HTL epitope, and all the CTL epitopes were predicted as IL-6 inducers. 7 B cell epitope, 6 HTL epitope, and 15 CTL epitope were predicted as inducing IL-10 which characterizes them with anti-inflammatory functions.

### 3.3 Construction of the multi-epitope vaccines

**Figure 1:**
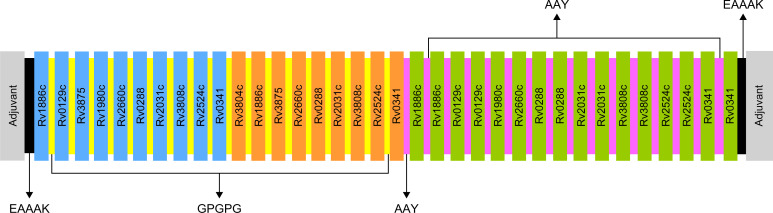
Schematic representation of the final multi-epitope vaccine peptide. Blue, orange, and green depict the designed vaccine construct with B-cell, HTL, and CTL epitopes. EAAAK linkers (black) were used to link the adjuvant with the N- and C-terminal sequences. B-cell and HTL epitopes were linked with GPGPG linkers (yellow), and CTL epitopes were linked with AAY linkers (purple).

Selected based on their higher antigenic and immunogenic properties as well as MHC class binding affinity, epitopes used for the multi-epitope vaccine candidates included 10 linear B-cell epitopes, 9 HTL epitopes, and 16 CTL epitopes. In addition, to augment the effectiveness of the multi-epitope vaccines, one of three adjuvants, including griselimycin (52, 53), HBD3 (54, 55), and 50sRP (56), were linked at the N- and C-terminals of the vaccine to produce three different vaccine constructs. Moreover, EAAAK, GPGPG, and AAY linkers were added between the adjuvant, B-, HTL, and CTL epitopes, respectively, to increase the accuracy of the expression of each peptide (**Figure 1**).

### 3.4 Antigenic, allergenic, toxicity, and physicochemical evaluation of the designed vaccines

The antigenicity of each vaccine candidate represents their ability to be recognized as antigens and their ability to advance immune responses. The antigenicity of each multi-epitope vaccine was predicted using VaxiJen 2.0, and those with a score >0.4 were considered to be antigenic. The griselimycin-, HBD3-, and 50sRP-TB vaccines showed scores of 0.7339, 0.7380, and 0.6375, respectively. Additionally, antigenicity was confirmed using ANTIGENpro, in which griselimycin-TB scored 0.791234, HBD3-TB scored 0.815016, and 50sRP-TB scored 0.873047. Based on the results from analysis using AllerTOP v2.0 and AllergenFP and ToxinPred, the multi-epitope vaccines were estimated to be non-allergenic and non-toxic (**Supplementary Table S1**).

The physicochemical properties of the multi-epitope vaccines were evaluated using Expasy ProtParam. Molecular weight of griselimycin-, HBD3, and 50sRP-adjuvanted vaccine candidates which are named as griselimycin-TB, HBD3-TB, and 50sRP-TB are 61kDa, 69kDa, and 85kDa in order. **Supplementary Table S1** shows that the theoretical pI values of the griselimycin- and 50sRP-TB models were 5.00 and 4.74, respectively, suggesting slight acidity, whereas that of the HBD3-TB model appeared to be slightly basic. The aliphatic index of the three vaccine models ranged from 67 to 76, indicating the thermostability of the vaccine constructs. The griselimycin-TB model has an estimated half-life of 100 h in mammalian reticulocytes, >20 h in yeasts, and >10 h in Escherichia coli. The estimated half-lives of the HBD3- and 50sRP-TB models are 30 h in mammalian reticulocytes, >20 h in yeasts, and >10 h in *E. coli*. The GRAVY scores were -0.202, -0.318, and -0.12 for the griselimycin-, HBD3-, and 50sRP-TB models, respectively, with negative GRAVY scores indicating that the structures are maintained under natural hydrophilic conditions and that they interact with water molecules. In addition, the instability indices of the griselimycin-, HBD3-, and 50sRP-TB models were 32.99, 34.25, and 28.18, respectively, suggesting they are stable, as an instability index >40 is considered unstable (**Supplementary Table S1**). Moreover, the solubility scores for griselimycin-, HBD3-, and 50sRP-TB were 0.302, 0.315, and 0.514, respectively (**Supplementary Figures S1Ai–Ci**). The whole sequences of griselimycin-, HBD3-, and 50sRP-TB models were estimated to have 33.3%, 30.4%, and 37.5% α-helix and 11.4%, 15.9%, and 13.0% β-strands (**Supplementary Figures S1Aii–Cii**).

Furthermore, a number of peptide-based vaccines and therapeutics for infectious diseases have received approval, and the strategic utilization of already sanctioned vaccines through matching or similarity queries within the THPdb database could lead to more auspicious outcomes (89). In this context, our research was directed specifically toward the THPdb database with an emphasis on data pertinent to contagious maladies such as tuberculosis. Unfortunately, we were unable to locate a specific peptide; only the PPD (Tuberculin Purified Protein Derivative) (ThPP ID as TH1194) could be retrieved. Additionally, we confirmed the potential of our multi-epitope model as a TB therapeutic strategy by validating the results using antiTBpdb (https://webs.iiitd.edu.in/raghava/antitbpdb/index.html) (90). Upon setting the E value in antiTBpdb at < 0.1, HTL and CTL epitopes of Rv0288 (TB10.4) and HTL epitope of Rv2031c (HspX) exhibited high similarities to the anti-tubercular peptides (**Supplementary Table S2**, The E values were in the following order: 4E-05, 0.037, 0.063). Therefore, there is potential for our model to be considered a therapeutic option, possibly after sequence modification.

### 3.5 Structural modeling, refinement, and validation of the multi-epitope vaccines

**Figure 2:**
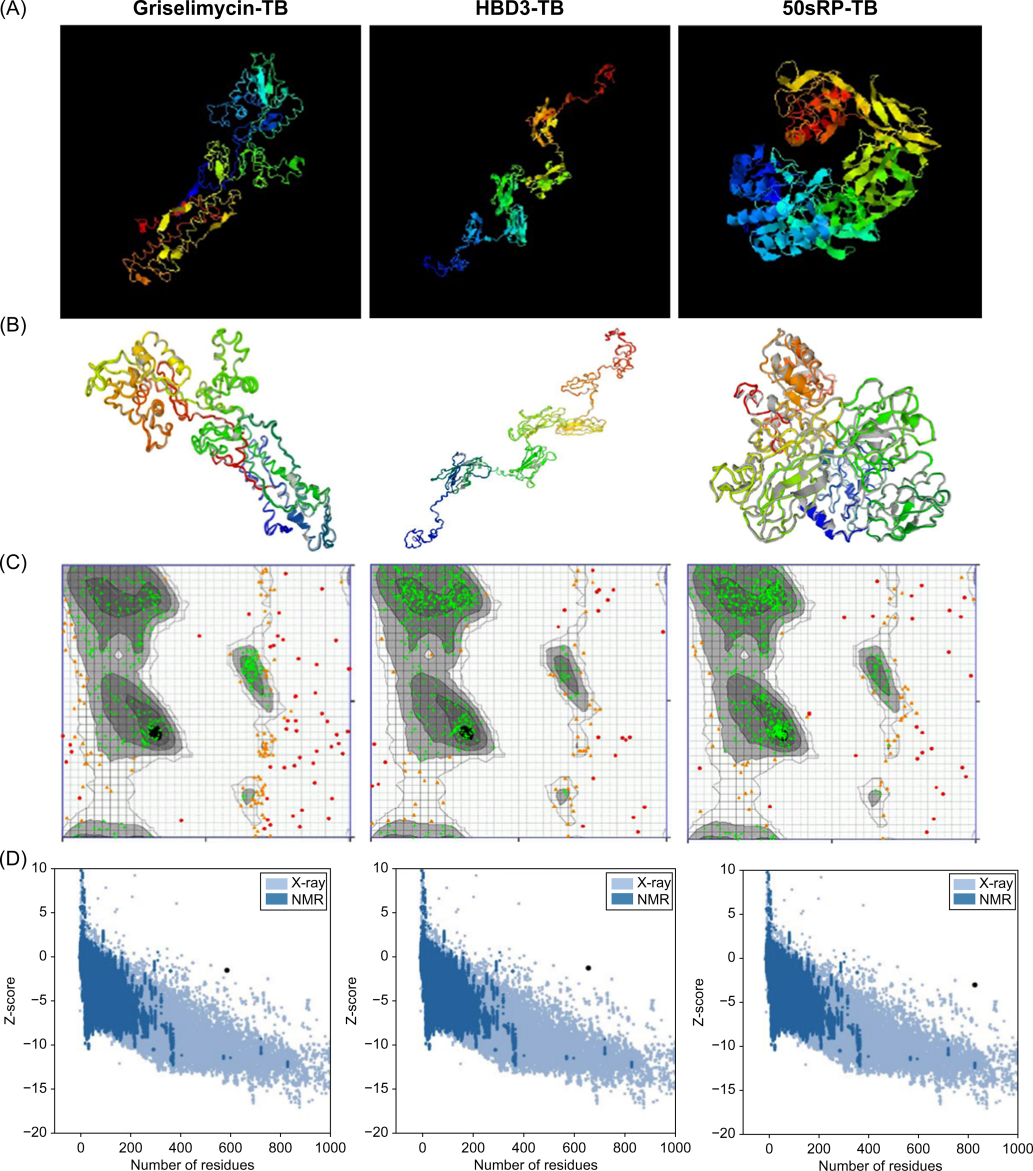
Protein 3D modeling, refinement, and validation. **(A)** Final 3D model of each multi-epitope vaccine depicted using I-TASSER. **(B)** Refinement of each construct was conducted using the GalaxyRefine web server. The tertiary structure was validated using the **(C)** Ramachandran plot and **(D)** ProSA web server.

The tertiary structure of each model was predicted using the I-TASSER server based on TM value, C-score, and RMSD. Typically, C-score values range from -5 to 2, with higher scores indicating a greater level of confidence (69). A TM value >0.5 indicates an accurate topology model, while a TM value <0.17 indicates random similarity. RMSD indicates the conformational stability of the protein complex (91). The five predicted models from the griselimycin-TB construct had C-score values ranging from -3.63 to -1.57. The griselimycin-TB model had an estimated TM value of 0.52 ± 0.15 and RMSD of 11.4 ± 4.5Å. The five predicted models from the HBD3-TB construct had C-score values ranging from -3.53 to -1.33, an estimated TM value of 0.55 ± 1.5, and an RMSD of 11.1 ± 4.6Å. Finally, the five predicted models from the 50sRP-TB construct had C-score values ranging from -4.3 to -1.47, an estimated TM value of 0.53 ± 0.15, and an RMSD of 12.0 ± 4.4Å. The highest C-score models from each vaccine were selected for further refinement and validation (**Figure 2A**).

The GalaxyRefine web server was used to identify the initial “crude” vaccine models. Model 1 from each construct, the one that showed the highest C-score value and TM-score >0.5, was the most reliable refined structure based on structural quality (**Figure 2B**). The refined model was validated using Ramachandran plot analysis, ERRAT, and the ProSA web server. In the Ramachandran plot analysis, 50sRP-TB had both the highest and lowest disallowed scores out of all three vaccine models (**Figure 2C**; **Table 6**). The quality and potential errors of the refined models were verified using the ProSA-web and ERRAT servers. A good model has a Z-score of -6.07 predicted using the ProSA-web and an accepted range >50 in ERRAT (92). Among these models, 50sRP-TB showed the most promising results through Ramachandran plot analysis, as well as the closest Z-score (griselimycin-TB: -1.4, HBD3-TB: -1.2, and 50sRP-TB: -2.9). Moreover, the overall quality factor predicted using the ERRAT server also indicated a good model (griselimycin-TB: 41.2, HBD3-TB: 75.2, and 50sRP-TB: 68.7) (**Figure 2D**; **Table 6**).

**Table 6:** Summary of 3D tertiary structure validation of the multi-epitope TB vaccine with various adjuvants.

Model	Ramachandran			Web-based tools	
	Highly preferred	Preferred	Disallowed	ProSA z-score	ERRAT
Griselimycin-TB	71.0	19.5	9.5	-1.4	41.2
HBD3-TB	86.5	9.6	3.9	-1.2	75.2
50sRP-TB	89.3	7.3	3.4	-2.9	68.7

### 3.6 Prediction of B-cell epitopes

**Figure 3:**
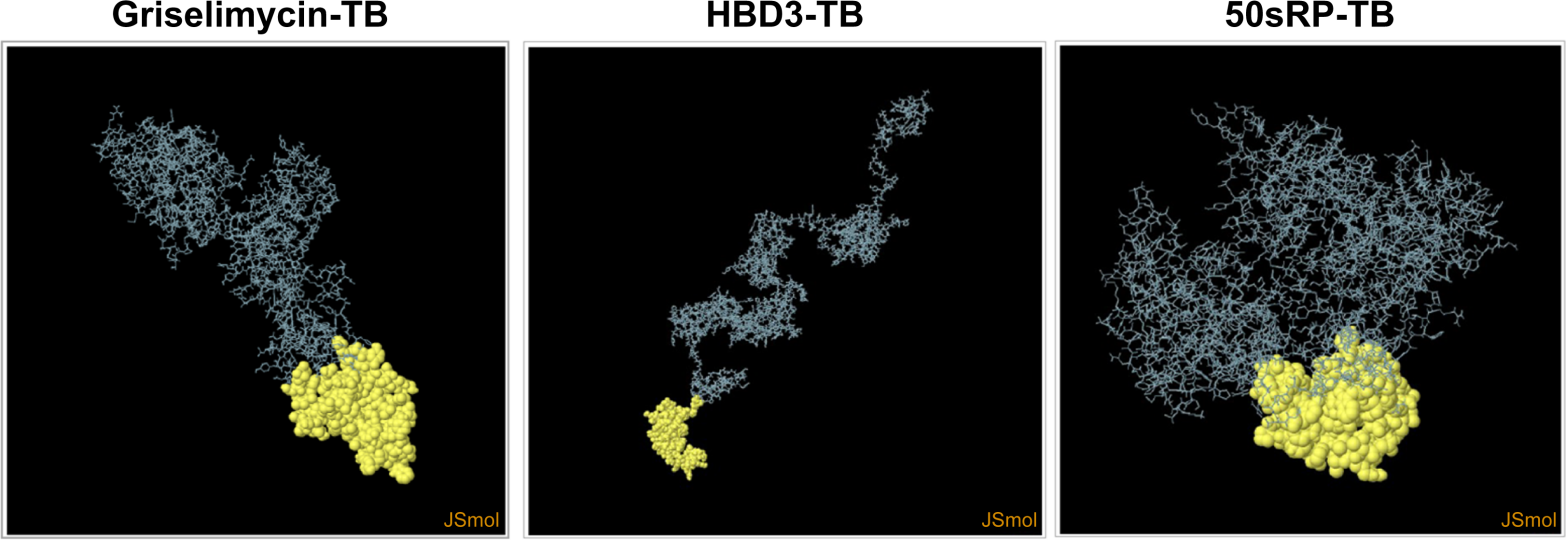
Representation of conformational or discontinuous B-cell epitopes of each designed multi-epitope vaccine. The conformational or discontinuous B-cell epitopes were represented with yellow circles, and the rest of the protein was represented with grey bars.

Structural epitope prediction was performed using the Ellipro tool from the IEDB server. For discontinuous peptide predictions, an AUC score of 0.69 or higher was selected. A total of 59 residues in griselimycin-TB, 48 in HBD3-TB, and 103 in 50sRP-TB were identified within discontinuous B-cell epitopes, with respective AUC scores of 0.787, 0.913, and 0.717 (**Figure 3** and **Table 7**).

**Table 7:** Predicted B-cell epitope residues of designed multi-epitope vaccines.

Model	Residues	Number of residues	Score
**Griselimycin-TB**	A:S277, A:V278, A:V279, A:G280, A:P281, A:G282, A:P283, A:G284, A:M285, A:S286, A:Q287, A:I288, A:M289, A:Y290, A:N291, A:Y292, A:P293, A:A294, A:M295, A:L296, A:G297, A:H298, A:A299, A:G300, A:P301, A:G302, A:P303, A:G304, A:A305, A:Y306, A:G307, A:S308, A:F309, A:V310, A:R311, A:T312, A:V313, A:S314, A:L315, A:P316, A:V317, A:G318, A:A319, A:G320, A:P321, A:G322, A:P323, A:G324, A:E325, A:L326, A:A327, A:A328, A:S329, A:S332, A:R333, A:V334, A:I335, A:L336, A:P337	59	0.787
**HBD3-TB**	A:E607, A:A608, A:A609, A:A610, A:K611, A:G612, A:I613, A:I614, A:N615, A:T616, A:L617, A:Q618, A:K619, A:Y620, A:Y621, A:C622, A:V624, A:R625, A:G626, A:G627, A:R628, A:C629, A:A630, A:V631, A:L632, A:S633, A:C634, A:L635, A:P636, A:K637, A:E638, A:E639, A:Q640, A:I641, A:G642, A:K643, A:C644, A:S645, A:T646, A:R647, A:G648, A:R649, A:K650, A:C651, A:C652, A:R653, A:K655, A:K656	48	0.913
**50sRP-TB**	A:K268, A:S269, A:Y270, A:R287, A:W288, A:E289, A:D290, A:D291, A:P292, A:Q293, A:P294, A:G295, A:W296, A:Y297, A:D298, A:G299, A:P300, A:G301, A:P302, A:G303, A:T304, A:Q305, A:P306, A:Q307, A:H308, A:T309, A:P310, A:V311, A:E312, A:P313, A:P314, A:V315, A:H316, A:D317, A:K318, A:P319, A:G320, A:P321, A:G322, A:P323, A:G324, A:V325, A:G326, A:K327, A:L328, A:I329, A:A330, A:N331, A:N332, A:T333, A:R334, A:V335, A:W336, A:V337, A:Y338, A:C339, A:G340, A:P341, A:G342, A:P343, A:G344, A:R345, A:V347, A:G358, A:N359, A:G360, A:P361, A:G362, A:P363, A:G364, A:S365, A:D366, A:P367, A:A368, A:Y369, A:P381, A:G382, A:P383, A:G384, A:V385, A:G386, A:V387, A:G388, A:V389, A:G390, A:T391, A:E392, A:Q393, A:N395, A:Y410, A:Y412, A:P413, A:A414, A:M415, A:L416, A:G417, A:H418, A:A419, A:G420, A:P421, A:G422, A:P423, A:G424	103	0.717

### 3.7 Molecular docking analysis and dynamics simulations

**Figure 4:**
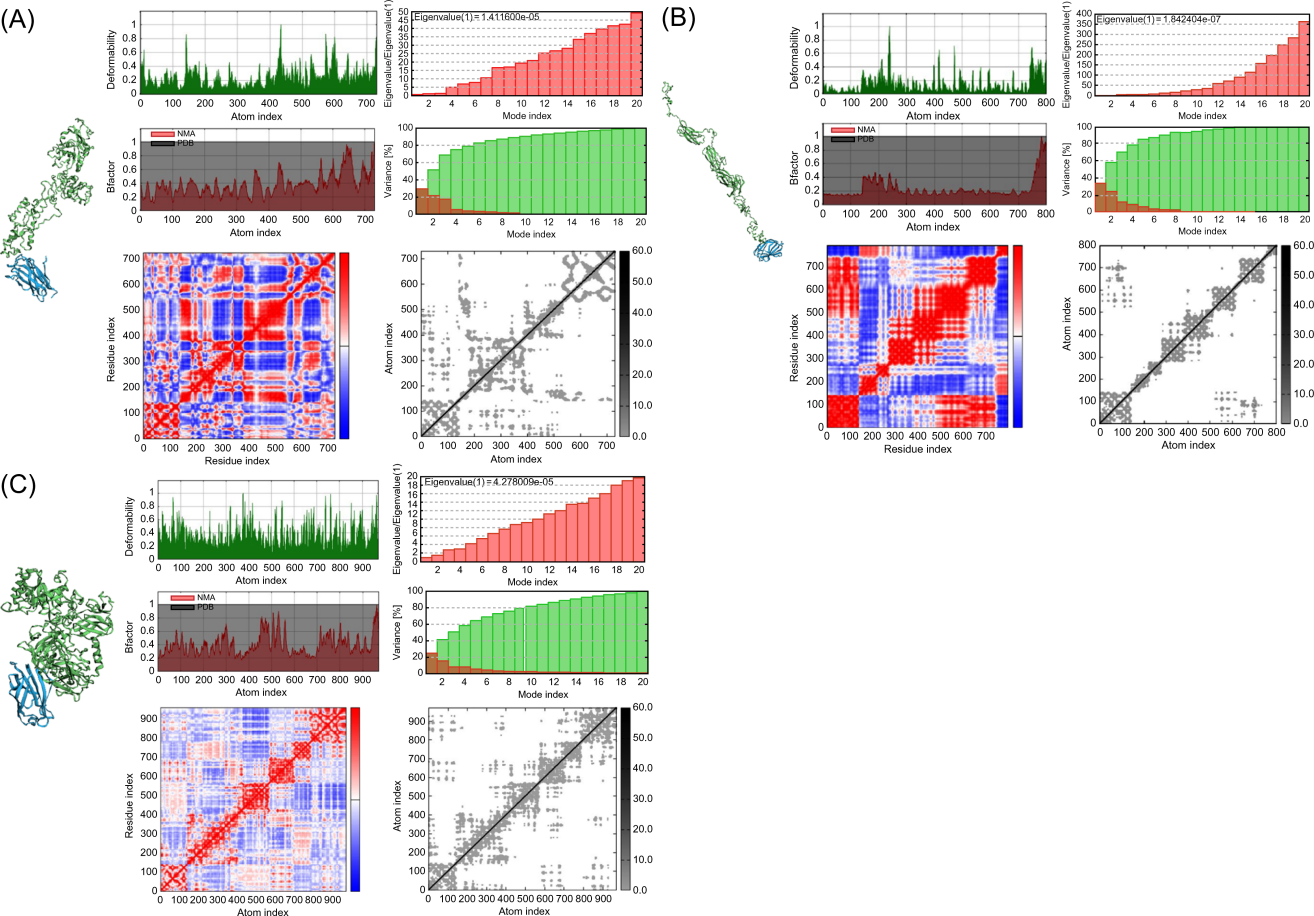
Normal mode analysis of vaccine constructs and their molecular docking with immune receptor TLR4. Normal mode analysis (NMA) mobility, deformability, eigenvalues, B factor, variance, co-variance map, and elastic network were evaluated in the vaccine constructs with the following adjuvants: **(A)** griselimycin, **(B)** HBD3, and **(C)** 50sRP.

To verify the immune response induced by interactions of the vaccines with TLRs, molecular docking analysis and dynamic simulations were performed using the HADDOCK server. TLRs are proteins expressed in target immune cells that recognize pathogen-associated molecular patterns to induce an innate immune response (93). Notably, TLR4 plays a crucial role in *M. tuberculosis* infection (94) and TLR4 agonist have been developed as vaccine adjuvant candidate for TB to increase the efficacy of the existing vaccine (95); therefore, the vaccine models were subjected to molecular docking analysis with TLR4 in the normal mode. Several parameters of the multi-epitope vaccine models and the TLR4-MD2 (PDB: 3FXI)-docked complex were evaluated using normal mode analysis (NMA) (**Figure 4**). The b-factor shows the relationship between the NMA and PDB of the docked model and represents the protein flexibility and mobility (96–98). The variance and covariance matrix provides the correlation between amino acid duplets and infers the most rigid models (99); a higher correlation means the better complex. Red indicates correlated residues; white indicates uncorrelated residues, and blue indicates anti-correlated residues (100). The elastic network shows the connecting matrix with rigid regions and classifies the atom pairs connected by a spring (101). Among the vaccine models, the 50sRP-TB model was identified as it includes several hinge regions, suggestive of the most deformative candidate. All models showed that the calculated b-factor was minimized compared to the native b-factor of PDB, implying minimization of the deformability of the structure. The eigenvalue of our vaccine models, griselimycin-, and 50sRP-TB was, respectively, 1.411600E-05, and 4.278009E-05, while that of TLR4 was 6.823114E-06 (**Figures 4A, C**). These findings show that docked complexes are stable with low deformation characteristics and have higher structural rigidity. For the griselimycin-, HBD3-, and 50sRP-TB vaccine models, the binding free energies were -44.64kcal/mol, -23.42kcal/mol, and -63.09kcal/mol, respectively.

Additionally, TLR2 and TLR3 play key roles in the innate immune response against *M. tuberculosis* infection (102–104). Based on docking analyses (**Supplementary Figure S2**), griselimycin-TB exhibited the highest binding affinity with TLR3, followed by TLR4 and TLR2. Similarly, HBD3-TB demonstrated the highest affinity for TLR4, TLR3, and TLR2, in that order. For 50sRP-TB, the highest binding affinity was observed with TLR3, followed by TLR2 and TLR4.

### 3.8 Codon optimization

**Figure 5:**
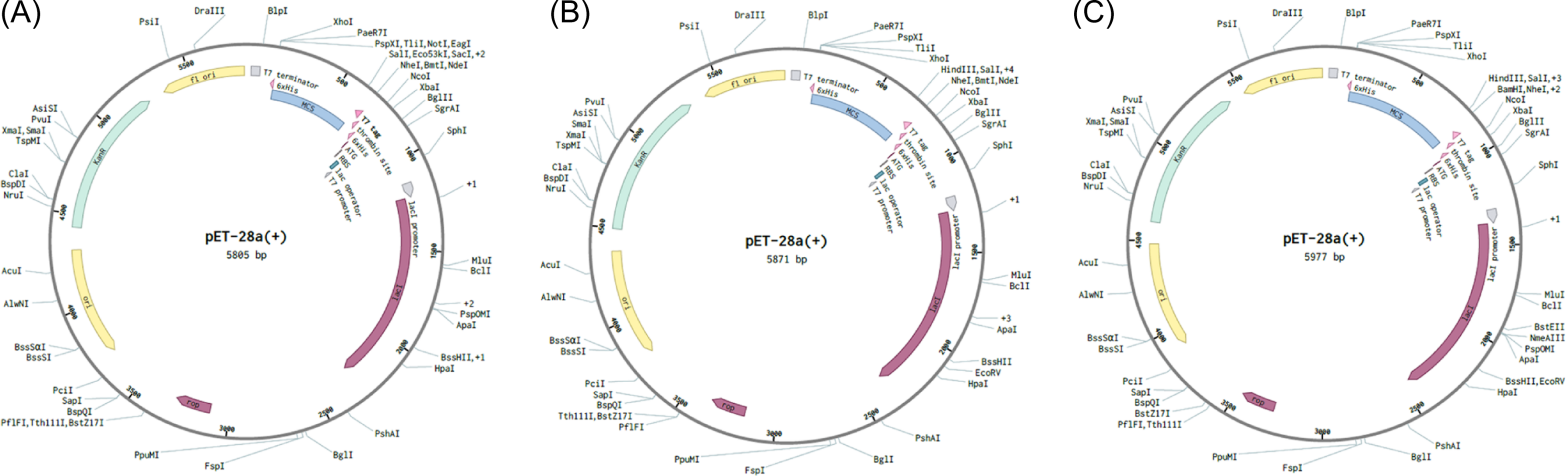
*In silico* restriction cloning of multiple-epitope vaccines into the expression vector pET30a(+). The codon sequence of each multi-epitope vaccine, **(A)** griselimycin-TB (7,020bp), **(B)** HBD3-TB (7,230bp), and **(C)** 50sRP-TB (7,740bp), was inserted in the multiple cloning site (MCS) of the pET30a(+) expression vector using the Snapgene sequence alignment tool.

The Java codon adaptation tool JCat was used with each of the respective multi-epitope vaccine models to optimize the codons for maximal expression in *E. coli* (strain K12). In codon-optimized sequences of the designed vaccines (griselimycin-, HBD3-, 50sRP-TB), the codon adaptation index values were 1.0, and the GC content values were 58.76%, 57.27%, and 55.25%, respectively. Additionally, the adapted codon sequences were optimized with sticky end restriction sites of *NdeⅠ* and *HindIII* at the N-terminus and C-terminus to facilitate restriction and cloning and inserted into the recombinant plasmid vector, pET-30a (+), using the Snapgene tool to design and effective cloning strategy (**Figure 5**).

### 3.9 Immune simulations

**Figure 6:**
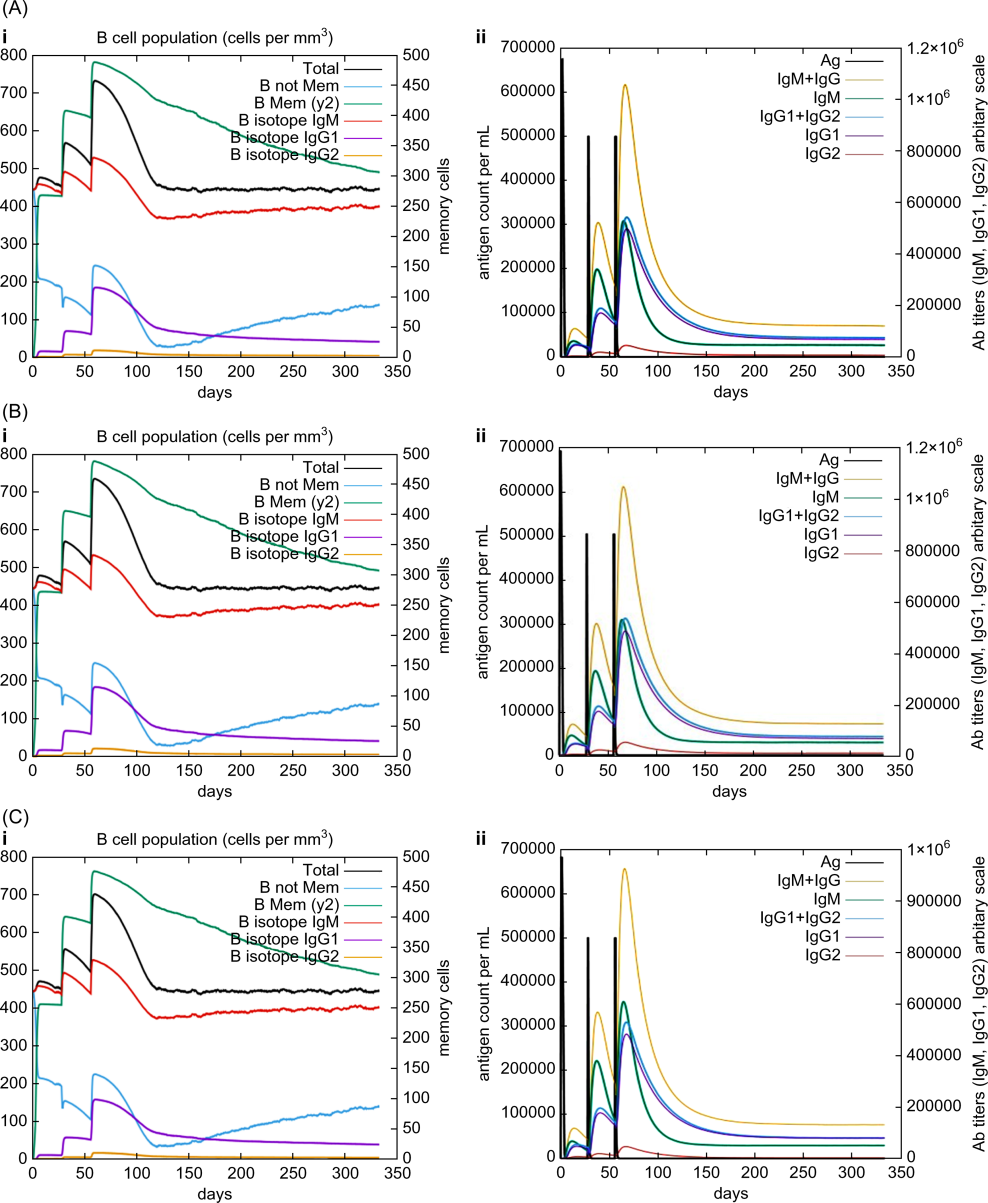
*In silico* C-ImmSim simulation of generated immune responses. Immunoglobulin production induced by multi-epitope vaccines with each respective adjuvant: **(A)** griselimycin, **(B)** HBD3, and **(C)** 50sRP. (i) The evolution of the B-cell populations and (ii) the production of various immunoglobulins in response to vaccination administered three times at twelve-week intervals.

**Figure 7:**
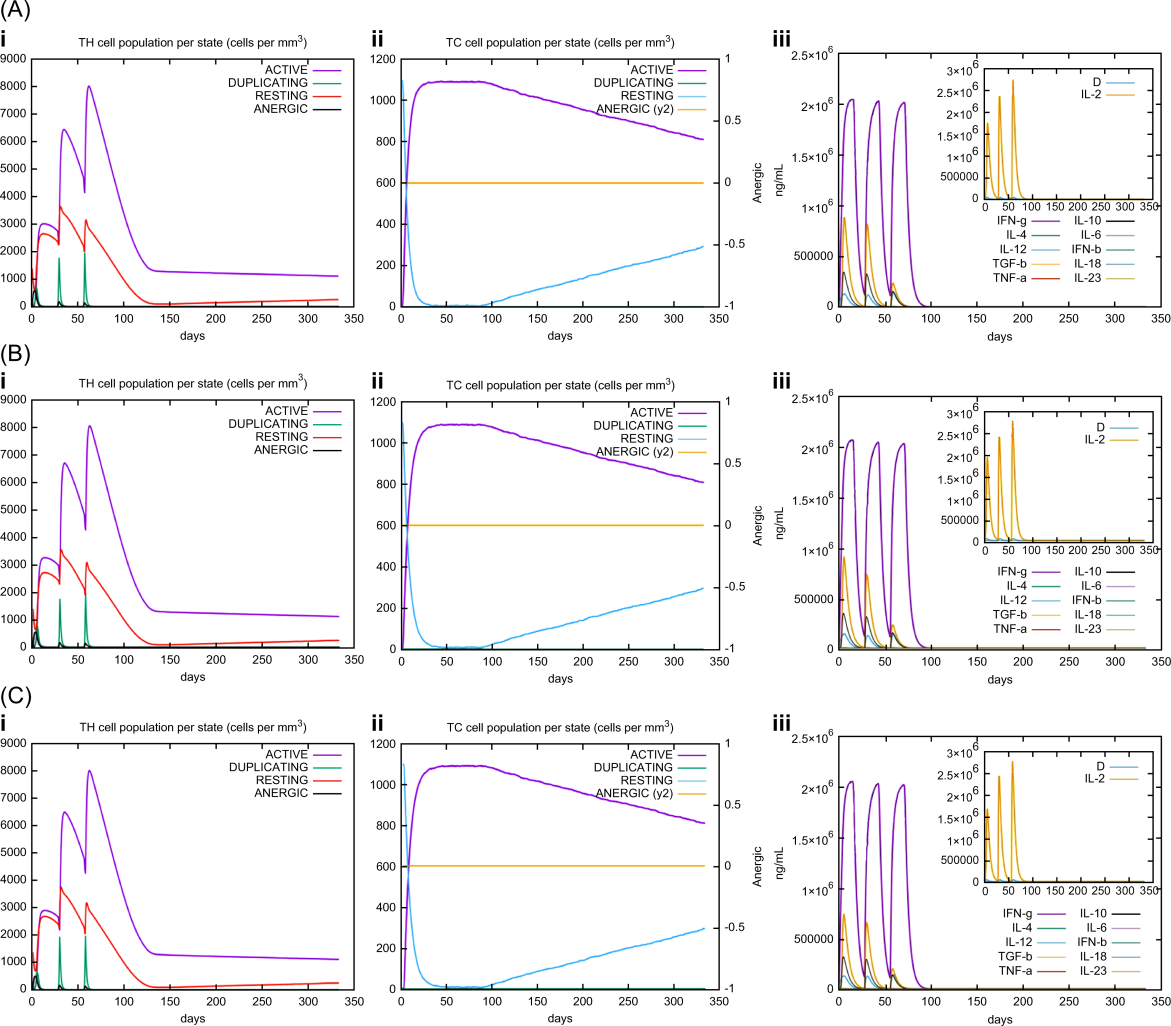
C-ImmSim simulation of the cytokine levels and antibody production. Predicted changes in (i) HTL population, (ii) CTL population, and (iii) cytokine levels in response to multi-epitope vaccines with respective adjuvants: **(A)** griselimycin, **(B)** HBD3, and **(C)** 50sRP.

*In silico* immune simulations were conducted using the C-ImmSim immune server to predict the immunological profile of the multi-epitope vaccines (**Figures 6**, **7**). The griselimycin-, HBD3-, and 50sRP-TB vaccine models elicited a significant increase in B cell population, including memory B cells, following repeated immunizations, indicating their capacity for isotype switching and memory cell formation (**Figure 6**). In **Figure 6**, antibody levels (IgM+IgG, IgG1+IgG2, IgM, and IgG) were found to increase during secondary and tertiary immunizations, accompanied by a decrease in antigen count. Furthermore, both CTL and HTL populations increased following secondary and tertiary immunization (**Figure 7**). Notably, a significant increase in IFN-γ production and a moderate increase in IL-2 were observed after the third vaccination. These data underscore the potential of our vaccine candidate to induce an effective immune response.

### 3.10 *In vitro* expression

To verify the expression of the vaccine model, we selected on candidate based on solubility and TLR4 docking model analysis. Predicting protein solubility is crucial for the selection of highly effective candidate proteins, as it can help avoid protein aggregation, which adversely affects biological activity and can lead to failures in the recombinant protein pipeline (105). Consequently, 50sRP-TB, which demonstrated the highest solubility score, was selected for expression in *E. coli*. Moreover, the 50sRP-TB model exhibited the lowest Gibbs free energy in the TLR4 docking model, suggesting its potential to induce an active TLR4-mediated immune response. We validated *in vitro* expression and purification using SDS-PAGE and western blot. The protein was best expressed in 16-h induction at 15°C in supernatant of cell lysate and molecular weight inclusive of His-tag molecular weight was approximately 80kDa (**Supplementary Figure S3**).

To verify the expression of the vaccine model, 50sRP-TB which showed the highest solubility score, was selected and expressed in *E. coli*. We validated *in vitro* expression and purification using SDS-PAGE and western blot. The protein was best expressed in 16-h induction at 15°C and molecular weight inclusive of His-tag molecular weight was approximately 80 kDa (**Supplementary Figure S3**).

### 3.11 Immunogenicity of vaccine candidate designed by *in silico* analysis

**Figure 8:**
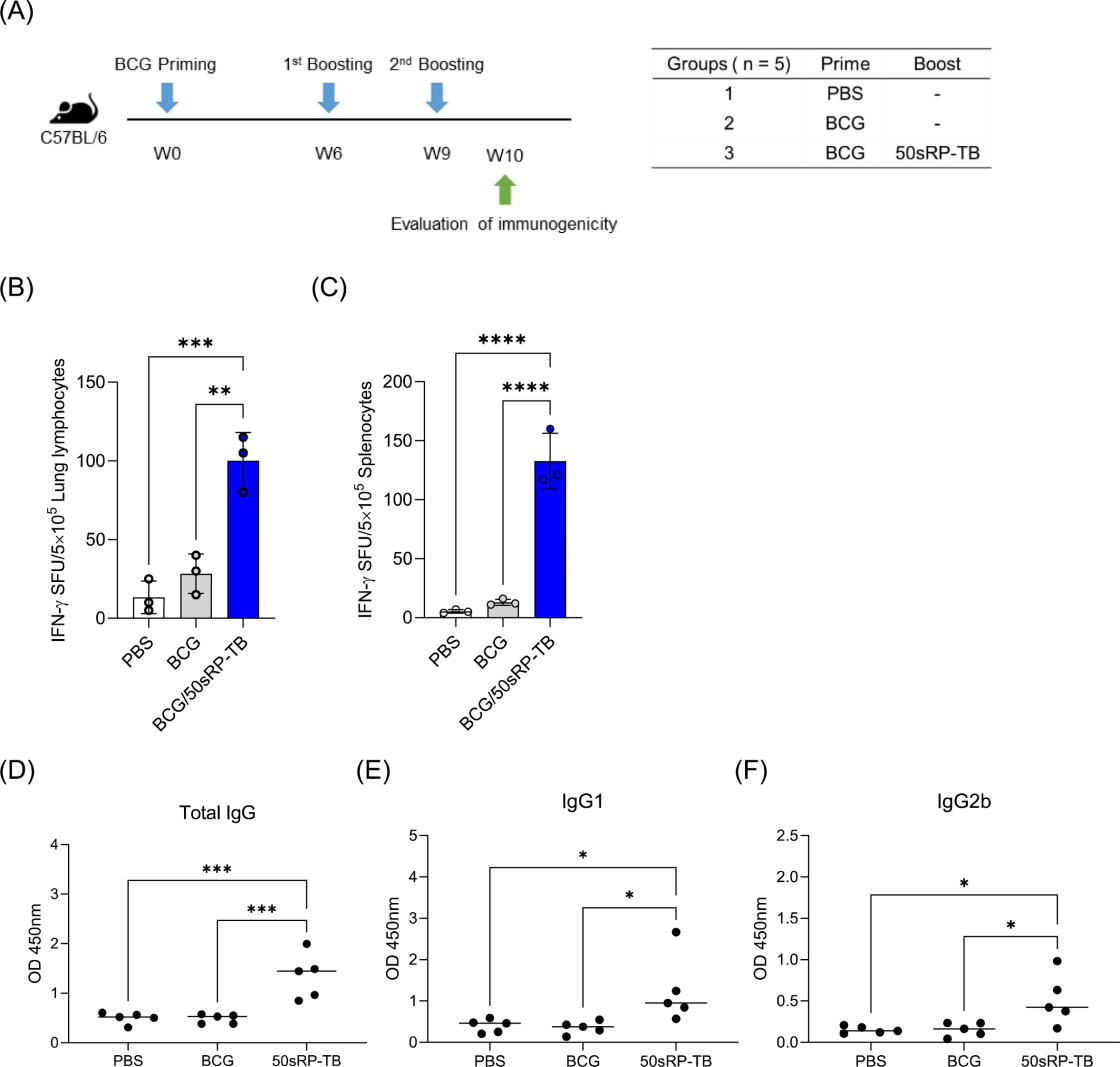
Immunogenicity of 50sRP-TB as a BCG-booster vaccine. **(A)** Schematic of the immunization schedule and subsequent evaluation. Mice (n = 5) were immunized by BCG 6 weeks before subunit vaccination. Subcutaneous immunization of 50sRP-TB was conducted and immunological analysis was assessed one week after the last immunization. Mice were immunized and euthanized as described in the methods section. Single cells stimulated with 50sRP-TB was detected interferon-γ (IFN-γ) secretion using an ELISpot assay in **(B)** lung lymphocyte and **(C)** splenocyte. **(D)** Antigen-specific total IgG, **(E)** IgG1, and **(F)** titer in serum was measured using ELISA. Data show the mean ± standard deviation from triplicate wells in each group; *p < 0.05, **p < 0.01, ***p < 0.001, and ****p < 0.0001 obtained using unpaired t-test.

To examine the effectiveness of artificially designed vaccine product, we investigated immunogenicity of 50sRP-TB in BCG-primed mouse. Mice were immunized subcutaneously with BCG and boosted with two doses of 50sRP-TB (**Figure 8A**). Immunologic analysis was performed with 50sRP-TB specific IFN-γ secretion and IgG, IgG1, and IgG2b measurement. Using ELISpot assay, multi-epitope peptides specific IFN-γ secreted cells were detected in lung lymphocytes and splenocytes. The number of IFN-γ spots in the BCG/50sRP-TB immunized group was significantly higher than those in the PBS or BCG only immunized groups, both in lung lymphocytes and splenocytes (**Figures 8B, C**). In addition, 50sRP-TB immunized mice generated higher titers of pooled multi-epitope peptides-specific IgG(H+L) and IgG1 than the other groups (**Figures 8D, E**). Remarkably, 50sRP-TB immunization elicited IgG2b production, which is associated with Th1 response (**Figure 8F**).

## 4 Discussion

TB remains a life-threatening disease, despite the presence of the approved BCG vaccine, as BCG shows limited protection against TB in adolescents and adults. Therefore, new vaccine candidates against TB are being studied and evaluated in clinical trials. Recently, the severity of COVID-19-TB co-infection was reported, along with its serious economic impact and hazard to global public health (106). Specifically, TB patients co-infected with SARS-CoV-2, showed severe symptoms caused by a “cytokine storm”, particularly within the lungs, heart, and liver (106–108). To prevent simultaneous infection with TB and COVID-19 studies on the development of vaccines against co-infection in several groups are ongoing (109).

The inherent complexities of tuberculosis present significant challenges to vaccine development. In the present study, we aimed to overcome such obstacles by designing a novel adjuvanted, epitope-based vaccine, grounded on antigens that have demonstrated efficacy in *in vivo* and clinical studies, with the goal of enhancing immunogenicity. Therefore, in this study, we have selected antigens with their vaccine efficacy validated through *in vivo* studies using animal models or clinical trials the virulence factor of *M. tuberculosis* with (24–34, 110). Also, we include the antigens GlfT2, Fas, and IniB, based on a previous antigen identification study (35). We predicted B- and T-cell epitopes using these selected antigens and designed peptide-based vaccines. Peptide-based vaccines have the advantage of improved immunogenicity due to their characteristic, aggregated immunodominant epitopes, and reduced side effects (22). The epitope sequences under consideration were distinctly demarcated by their potential to modulate inflammatory responses, either amplifying or mitigating the cascade. By leveraging a comprehensive set of immunoinformatics methodologies, the modulation capacity of each epitope sequence was determined for cytokines, IL-4, IL-6, and IL-10. From this analysis, it was inferred that six B-cell epitopes, five HTL epitopes, and twelve CTL epitopes could potentially serve as inducers of IL-4 secretion. In juxtaposition, eight B-cell epitopes, one singular HTL epitope, and sixteen CTL epitopes were projected to function as inducers of IL-6 secretion. Taken together, a notable fraction of the CTL epitope sequences appeared to be predisposed as inducers for both IL-4 and IL-6, cytokines intrinsically associated with the regulatory mechanisms underlying *M. tuberculosis* infection. Notably, only the HTL epitope derived from Rv3808c (GlfT2) was validated as an inducer for IL-10, a cytokine representative of the anti-inflammatory prowess in mycobacterial disease dynamics.

In this study, vaccine candidates were linked epitope sequences with AAY and GPGPG linkers to express respective antigens with reduced junctional immunogenicity (111). In mammalian cells, the AAY (Ala-Ala-Tyr) linker acts as the cleavage site and multi-epitope vaccines assembled with the AAY linker showed enhanced epitope presentation and structure stability (112). The Glycine-rich linker, GPGPG, was designed as a universal spacer and is known to induce the HTL immune response (113, 114). An EAAAK linker incorporated between epitopes and adjuvants improves the bioactivity of the fused proteins and increases the expression level and stability of the vaccine construct (115).

Several bioinformatics tools were employed to assess physicochemical properties, antigenicity, allergenicity, and solubility for epitope-based vaccine design. Expasy ProtParam computes various physicochemical characteristics derived from a protein sequence without additional information. This tool calculates both pI and MW and forecasts amino acid composition, atomic composition, extinction coefficient, estimated half-life, and instability index, among other parameters (116). According to the GRAVY score, which assesses maintenance ability in hydrophilic or hydrophobic environments, all three models displayed negative GRAVY values, suggesting a higher structural stability in a hydrophilic environment. This aspect can be correlated with solubility, critical in determining *in vitro* protein expression. To enhance immunogenicity, we designed and forecasted vaccine properties involving adjuvants such as griselimycin, HBD3, and 50sRP. As an adjuvant sequence was added into the N- and C-terminus of the epitope sequence, its protein solubility was predicted to be higher than that of the original sequence (although data are not included, the solubility of the original sequence was 0.292).

Furthermore, all models were considered both stable and thermostable. The prediction of secondary and tertiary structures of the target proteins is essential in vaccine development to induce an immune response (117). The 3D structure of our multi-epitope models showed desirable stability after all refining processes and appropriate characteristics based on the results of the Ramachandran plots. Furthermore, based on the Ramachandran plots, which showed that the residues were present in the favorable regions (118), the 50sRP-TB model was the most acceptable.

TLRs is expressed in monocyte, immature DC and macrophage cells (119) and binds to several antigens derived from *M. tuberculosis* of pathogen itself, resulting development of TLRs targeted vaccine candidates and adjuvants (120). In this study, TLR binding affinity and dynamic simulation of each model were used to predict their immune response induction capacity. The conformational changes in the TLR molecule upon binding the vaccine suggested that the complex can process downstream signal cascades (99). To evaluate conformational changes of vaccine candidate and TLR complex, protein flexibility was examined by the NMA study through various analyzed results. A greater part of the complex individual chains showed higher rigid regions in all models. Binding affinities of the TLR4-MD2 complex-, TLR2-, and TLR3-vaccine-docked complexes were predicted. The binding affinity of the vaccines to the TLR4-MD2 complex, particularly the 50sRP-TB model, suggests significant induction of innate and adaptive immunity. Additionally, vaccine binding affinity with TLR2 and TLR3, which play a crucial role in *M. tuberculosis* protection following infection, demonstrated that all models elicit a good immune response.

In the context of TB vaccine research, it is imperative to underscore the dual necessity of prevention and therapeutic intervention. Notably, there exists a clinical phenomenon where patients who have ostensibly recovered from TB witness a reactivation or relapse of latent TB. Given this clinical challenge, the emphasis on developing therapeutic vaccines has garnered significant attention. Stemming from this perspective, certain vaccine candidates, derived from foundational research, have showcased both prophylactic and therapeutic potential, subsequently progressing to the clinical evaluation phase, and prominent among these are ID93/GLA-SE, H56:IC31, and VPM1002 (121).

The findings of the present study suggest that vaccine designed with multiple epitopes of TB are potential candidates, as one of the candidates, 50sRP-TB, exhibited soluble characteristic in *E. coli* and immunogenic properties in a mouse. Although there are no distinct immune correlates of protection, CD4^+^ T cell response, especially IFN-γ, is one of the important indicators in TB vaccine (122). Based on immunogenicity evaluation, 50sRP-TB elicited IFN-γ secreted T cell response and IgG2b production which is engaged in Th1 response in BCG-primed mice. This result implies that 50sRP-TB is a promising T cell response-inducible vaccine. Moreover, we anticipate protective effectiveness of 50sRP-TB in a *M. tuberculosis* challenged mouse in the further study.

Although our candidates showed good protein characteristic and immunogenicity, our study had a limitation. Since the vaccine candidate was designed using human MHC epitopes, its effectiveness needs to be evaluated with a humanized mouse model. G.W. et al. reported the reliable immunogenicity and protective efficacy of MP3RP, designed *in silico* in the lungs and spleen of a humanized mouse model to overcome the limitation posed by differences in human and mouse MHC allele (123). However, they also have several drawbacks when evaluating vaccine efficacy. Due to the distinction in antigen presentation between murine MHC (H-2) and human MHC (HLA), an MHC-humanized mouse model has been recently adopted in research to evaluate MHC-restricted epitope-based vaccines (110, 123, 124). Based on these studies, going forward, for the development of a preventive and therapeutic vaccine for TB, the development of a preventive and therapeutic vaccine for TB, this proposed vaccine will be validated for its efficacy in *in vivo* studies in a suitable mouse model.

## Data Availability

The original contributions presented in the study are included in the article/**Supplementary Material**. Further inquiries can be directed to the corresponding author.
