# Supplementary material for: In silico analysis for the development of multi-epitope vaccines against Mycobacterium tuberculosis
Source: Front Immunol. 2024 Nov 18;15:1474346. doi: 10.3389/fimmu.2024.1474346 (PMC11609213; doi:10.3389/fimmu.2024.1474346)
Supplement: Supplementary file 1 [file DataSheet1.docx]

Supplementary Material

***In silico* analysis for the development of multi-epitope vaccines against *Mycobacterium tuberculosis***

**Jin-Seung Yun^1,3^, A Reum Kim^2^, SooMin Kim^1^, Eunkyung Shin^1^, Sang-Jun Ha^3^, Dokeun Kim^1^, and Hye-Sook Jeong^1*^**

*** Correspondence:** Hye-Sook Jeong: [jeongnih@korea.kr](mailto:jeongnih@korea.kr)

# Supplementary Figures and Tables

- 1. Supplementary Tables

**Supplementary Table S1**. Various physicochemical properties of multi-epitope TB vaccines with three different adjuvants.

|  | | **Griselimycin-TB** | **HBD3-TB** | **50sRP-TB** |
| --- | --- | --- | --- | --- |
| **Molecular Weight (kDa)** | | 61 | 69 | 85 |
| **Toxicity** | | Non-toxic | Non-toxic | Non-toxic |
| **Allergenicity** | | Probable Non-allergen | Probable Non-allergen | Probable Non-allergen |
| **pI** | | 5.00 | 8.76 | 4.74 |
| **Aliphatic index** | | 67.92 | 64.54 | 76.61 |
| **Half-life** | Mammalian reticulocyte  (*in vitro*) | 100 hours | 30 hours | 30 hours |
|  | Yeast (*in vivo*) | >20 hours | >20 hours | >20 hours |
|  | Escherichia coli (*in vivo*) | >10 hours | >10 hours | >10 hours |
| **GRAVY score** | | -0.202 | -0.318 | -0.12 |
| **Instability index** | | 32.99 | 34.25 | 28.18 |

pI: isoelectric point, GRAVY: grand average of hydropathy.

**Supplementary Table S2.** Similarity to epitope sequences of the vaccine model and anti-tubercular or anti-mycobacterial peptides.

|  | **Sequence** | **Sequences producing significant alignments** | **Score (bits)** | **E value** |
| --- | --- | --- | --- | --- |
| Rv0288 HTL epitope | MSQIMYNYPAMLGHA | Antitb_1462 (QIMYNYPAM) | 22 | 0.037 |
|  |  | Antitb_1459 (QIMYNYPAM) | 22 | 0.037 |
|  |  | Antitb_1463 (IMYNYPAML) | 22 | 0.044 |
|  |  | Antitb_1450 (IMYNYPAML) | 22 | 0.044 |
|  |  | Antitb_1439 (IMYNYPAML) | 22 | 0.044 |
| Rv2660c CTL epitope | AMEDLVRAY | Antitb_1464 (AMEDLVRAY) | 21 | 0.063 |
| Rv2031c HTL epitope | AYGSFVRTVSLPVGA | Antitb_1267 (AYGSFVRTVSLPVGA) | 32 | 4E-05 |
|  |  | Antitb_1266 (AYGSFVRTVSLPVGA) | 32 | 4E-05 |
|  |  | Antitb_1650 (AYGSFVRTVSLPV) | 28 | 4E-04 |
|  |  | Antitb_1649 (AYGSFVRTVSLPV) | 28 | 4E-05 |
|  |  | Antitb_1222 (AYGSFVRTVSLPV) | 28 | 4E-05 |

- 1. Supplementary Figures

**
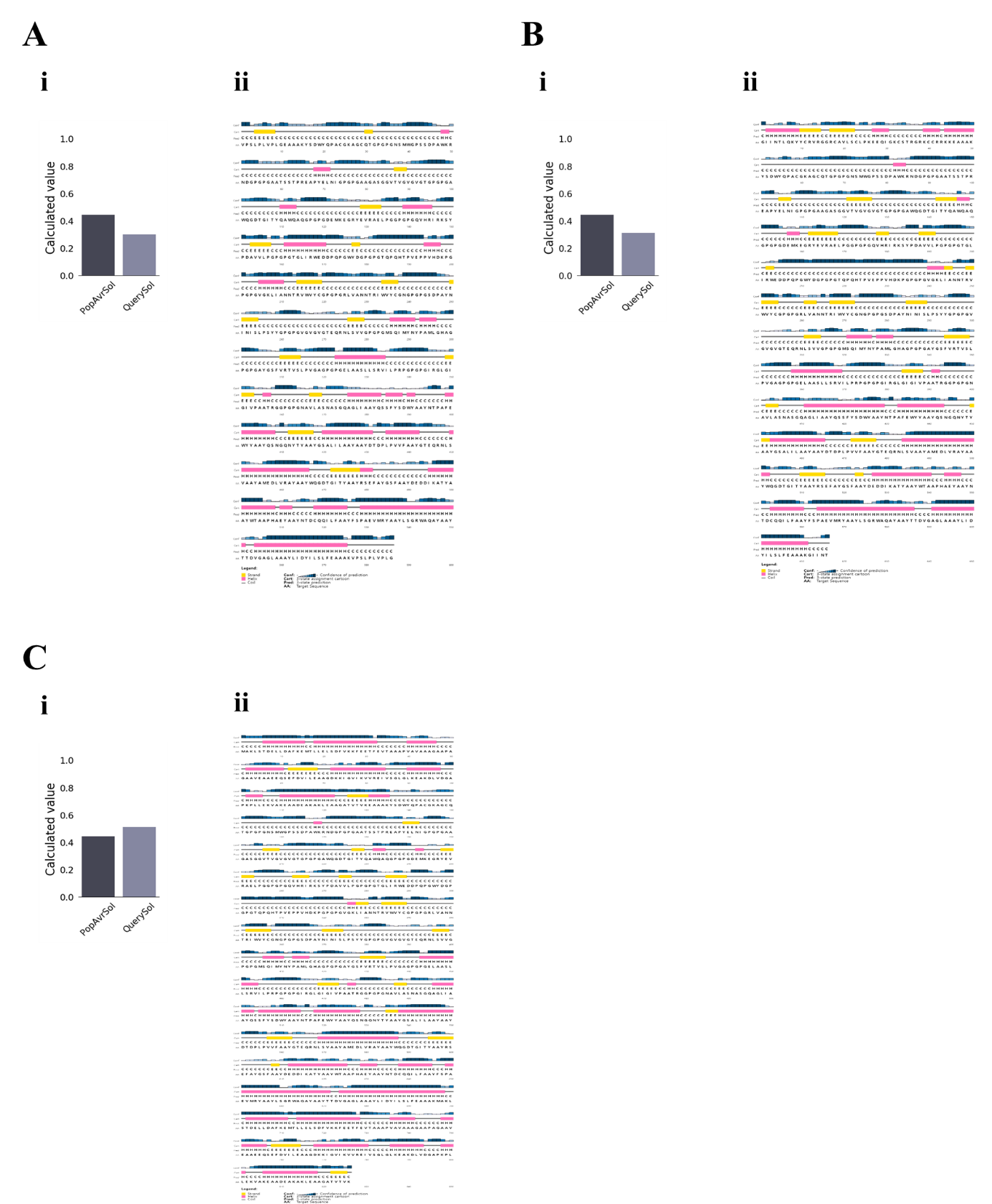
**

**Supplementary Figure S1. Graphical representation of secondary structure features and solubility analysis of the final subunit vaccine sequence.** Secondary structure predictions and solubility analyses of multi-epitope TB vaccine constructs using **(A)** griselimycin, **(B)** HBD3, and **(C)** 50sRP as adjuvants were conducted with PRISPRED, Expasy ProsParam, and Protein-Sol servers.

**
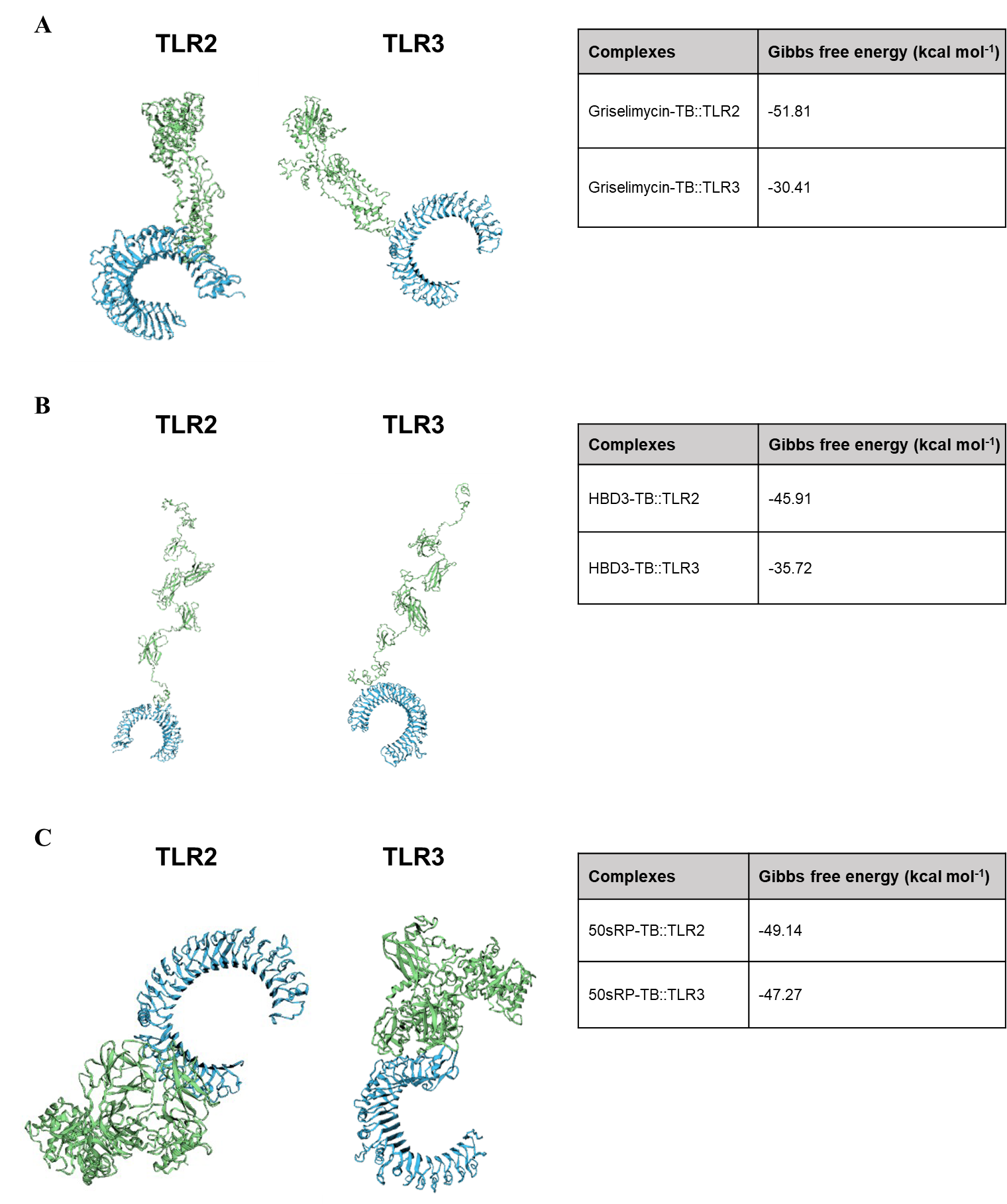
**

**Supplementary Figure S2.** Molecular docking of multi-epitope TB vaccine constructs using (**A)** griselimycin, (**B)** HBD3, and **(C)** 50sRP as adjuvants with immune receptors TLR2 and TLR3.


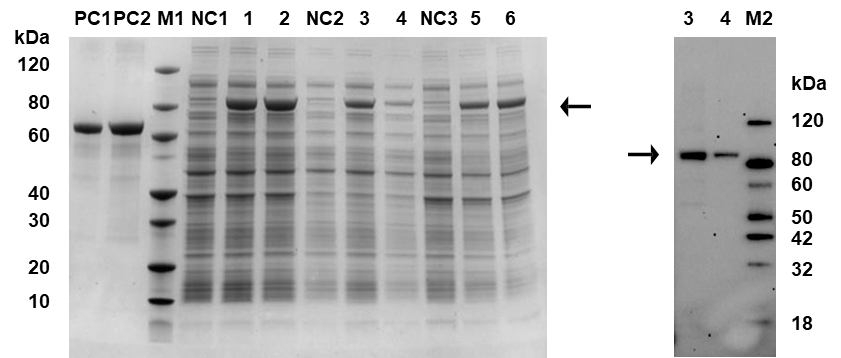


**Supplementary Figure S3.** SDS-PAGE (left) and western blot analyses (right) that validate *in vitro* expression of 50sRP-TB. M1: Protein marker, M2: Western blot marker, PC1: BSA (1μg), PC2: BSA (2μg), NC1: Cell lysate without induction, 1: Cell lysate with induction for 16 h at 15℃, 2: Cell lysate with induction for 4 h at 37℃, NC2: Supernatant of cell lysate without induction, 3: Supernatant of cell lysate with induction for 16 h at 15℃, 4: Supernatant of cell lysate with induction for 4 h at 37℃, NC3: Pellet of cell lysate without induction, 5: Pellet of cell lysate with induction for 16 h at 15℃, 6: Pellet of cell lysate with induction for 4 h at 37℃.
